# Development and assessment of vibrotactile feedback from the embedded sensors of a microprocessor-controlled knee prosthesis

**DOI:** 10.1186/s12984-025-01793-8

**Published:** 2025-12-19

**Authors:** Romain Valette, Jose Gonzalez-Vargas, Strahinja Dosen

**Affiliations:** 1 Department of Health Science and Technology, Aalborg University, Aalborg, Denmark; 2 Ottobock SE & Co. KGaA, Duderstadt, Germany

**Keywords:** Artificial sensory feedback, Lower-limb amputation, Vibrotactile stimulation, Microprocessor-controlled prosthesis, Prosthesis, Gait, Overground walking, Stair ambulation, User experience, User preferences

## Abstract

**Background:**

Artificial sensory feedback can improve function and user experience in lower-limb prosthesis users. Non-invasive methods like vibrotactile stimulation are clinically convenient, as they require no surgery. Most studies evaluate single feedback approaches, typically under controlled conditions promoting reliance on feedback. This study presents a flexible framework to compare multiple feedback approaches using microprocessor-controlled prosthesis (MP) sensors during daily-life activities.

**Methods:**

Ten able-bodied participants and one prosthesis user with transfemoral amputation (TFA) tested two feedback locations (waist “Belt”, or thigh/residual limb “Socket”) to investigate tradeoffs between perception quality and compactness, using Sensation Thresholds (ST), Weber Fraction (WF), Spatial Discrimination (SD), and comfort. TFA then completed an out-of-the-lab walking session with the Socket configuration to evaluate the impact of four feedback approaches on spatiotemporal parameters and kinematics symmetries, cognitive load, and user experience during overground walking and stair climbing. Three approaches used embedded MP sensors, conveying (1) knee angle, (2) hybrid (gait phases overground, knee angle during stairs), and (3) damping (velocity-dependent resistance to flexion/extension) feedback. The fourth method used a sensorized insole, providing (4) force feedback (plantar pressure under the prosthetic foot).

**Results:**

Able-bodied participants perceived the Belt configuration better–lower ST (29.09 ± 0.60% vs. 33.19 ± 0.60%, p < 0.001), lower WF (14.49 ± 7.02% vs. 17.98 ± 5.72%, p < 0.01), better SD at higher task difficulty (four choices: 99.3 ± 2.0% vs. 91.5 ± 2.0%, p < 0.01; eight choices: 96.0 ± 2.0% vs. 78.1 ± 2.0%, p < 0.001)–and found it also more comfortable (9.17 ± 0.3 vs. 8.15 ± 0.3; p < 0.05). Similar trends were observed in TFA. Feedback did not impact the kinematics symmetry but slightly affected stance time/percentage symmetry, with force feedback demonstrating the most consistent benefits. These suggest that incidental feedback provided intrinsically by the prosthesis (e.g., motion, sound, socket pressure, vibration) may already support gait in experienced users. Nevertheless, TFA preferred having feedback, especially force and damping, which reduced cognitive load.

**Conclusion:**

Embedded MP sensors enable flexible, compact feedback solutions, combining internal signals (e.g., damping feedback) with external sensing (e.g., omnidirectional force feedback). Belt-mounted vibromotors are effective for testing complex encoding schemes. Feedback should be co-developed with users, balancing objective performance and subjective experience.

**Supplementary Information:**

The online version contains supplementary material available at 10.1186/s12984-025-01793-8.

## Background

The occurrence of a lower limb amputation represents a profoundly distressing event, resulting in substantial functional, psychological, and cognitive impairments that affect individuals' capacities in performing everyday activities [1–3]. Lower-limb prostheses users express multifaceted needs, closely related to these impairments [4], and research has been focusing on improving the control and/or feedback of those devices [5, 6]. One step towards better performance was to develop microprocessor-controlled (MP) ankle and knee prostheses, which are systems that are electronically controlled to modulate the behavior of the prostheses and thereby make the gait more physiological and stable. The development of such systems has shown to be widely beneficial regarding the overall function (e.g., walking overground, ascending/descending stairs) and cognitive demands [7–10], yet the users still face suboptimal functional capacities and lower confidence [11, 12]. Therefore, this indicates that further aspects must be considered, and providing additional sources of feedback has been used as one approach to improve the ambulation and user experience.

The main idea when restoring feedback is to read the sensor data from the prosthesis or external instruments and sensors, and then translate this information into stimulation profiles delivered to the user invasively or non-invasively by stimulating peripheral nerves [5, 13–17] or skin of the residual limb [6, 18–20] respectively. So far, a wide variety of feedback solutions have been presented relying on information from instrumented treadmills and force platforms, sensorized insoles, inertial measurement units (IMUs), and electromyography (EMG) sensors, but also embedded sensors from MP prostheses [5, 6]. While providing feedback from the embedded sensors of a prosthesis is common in upper-limb prosthetics research [5, 21–26], only a few authors have proposed a similar approach in lower-limb prosthetic devices [13–17, 27]. Invasive feedback implementations have been explored, for example, by conveying knee angle information measured by the encoder of an MP knee (Rheo Knee XC, Össur, Iceland) through the modulation of the stimulation amplitude delivered via four TIME-4H electrodes implanted inside the sciatic nerve [14–17]. This stimulation elicited a tactile sensation around the posterior shank area and/or the contraction of the gastrocnemius or soleus muscles. Similarly, in a study employing an agonist-antagonist myoelectric interface (AMI), a functional electrical stimulator directly encoded torque information measured by the prosthesis [13]. In contrast, non-invasive feedback solutions using embedded sensing have received far less attention. A notable example is the work presented in [27], which combined embedded sensing with non-invasive vibrotactile feedback in a research-grade powered prosthesis, but in the context of teleoperation.

Modern MP prostheses are equipped with a wide range of embedded sensors to control their behavior, including not only knee and ankle joint encoders but also IMUs, as well as load cells [28]. These sensors enable the collection of comprehensive real-time gait data, including joint angle, angular velocity and angular acceleration, limb orientation, loads, and estimated gait phases [29], thereby offering significant potential for advanced feedback strategies and clinical gait analysis [30]. However, most feedback solutions for lower-limb prostheses have so far focused on a limited subset of these signals, and the full potential of embedded sensor data for user feedback-especially through non-invasive means, remains underexplored. This is an important goal since non-invasive feedback methods are simpler to translate into clinical applications [31, 32], as they avoid surgical risks and can be rapidly integrated into routine care, making them particularly promising for real-world use beyond the laboratory setting. Furthermore, using the sensors already integrated inside the prosthesis leads to a self-contained system.

The most common methods to deliver stimulation non-invasively are electrical impulses and miniature vibromotors. The advantage of electrical stimulation is that it is compact and can integrate many stimulation points, but the parameters need to be adjusted carefully (otherwise, uncomfortable and even painful sensations can be produced) [20, 33]. Vibromotors are more challenging to integrate but they are easy to apply. In both cases, however, the placement of stimulation elements (motors and electrodes) is an important factor when designing feedback. For instance, due to high pressures between the socket and the residual limb, the feedback components may cause discomfort, or the pressure and change in pressure may alter the perception of the feedback provided. Moreover, the motors and electrodes need to be placed to produce clear and consistent sensations, especially considering that the users’ perception seems to be altered when they start walking [33]. For these reasons, in addition to placing the stimulation channels inside the socket [34–40], which would be the most natural approach, the authors also investigated other feedback locations, such as inside a belt [41], proximally to the socket [18–20, 42–46], on the forearm [47, 48], hand [49], abdomen [41, 50, 51], lower back [48, 52], and even tongue [53]. A notable example of using multiple vibromotors on the thigh, though not relying on embedded sensors, is the system presented in [40], which aimed to restore sensory feedback from the insole in able-bodied participants using a bypass knee prosthesis. Among different placements, the belt configuration presents a promising tradeoff: it is easy to don, avoids potential interference with the socket, and allows for even spatial distribution of multiple motors around the waist, thereby maximizing the distance between the motors and potentially facilitating their perceptual localization and discrimination. Unlike more distal placements such as the forearm or the hand, the belt preserves anatomical proximity and consistent spatial mapping, which allows for the intuitive interpretation of the feedback [54]. The belt location also avoids the impact of residual limb movement and socket pressure on vibrotactile sensations [55–57]. However, a direct comparison between feedback delivered inside the socket and when using a belt in dynamic conditions has not been conducted.

In the present study, we developed a framework that allows quick design and testing of different feedback interfaces by exploiting the embedded sensing flexibility of an MP prosthesis. First, we conducted a psychophysical evaluation of the system, involving one participant with a transfemoral amputation and ten able-bodied participants walking with a bypass device. This method allows able-bodied participants to walk with the MP prosthesis and mimic the conditions of the socket interaction during gait with a prosthetic device [58]. This part of the experiment assessed perception capacities and subjective experience across two feedback configurations, where vibromotors were either placed inside the socket (around the thigh/residual limb) or positioned around the waist. Then, contrary to the usual approach in the literature, where a single feedback scheme is selected a priori and tested, we exploited the framework to implement a more exploratory (user-oriented) assessment where we compared the effect of four different feedback methods in the participant with transfemoral amputation. Although the feedback schemes were predefined by the research team, their assessment incorporated a range of objective (i.e., gait parameters during overground and stair walking) and subjective (i.e., perceived usefulness, comfort, and cognitive load) outcome measures. This structured involvement of the participant in assessing multiple aspects of each feedback modality and then ranking them according to their preference reflects a user-oriented approach, where user input directly informs the interpretation and future direction of feedback system design.

Three feedback methods used embedded information from the prosthesis whereas the last one employed an externally placed pressure insole [59]. The latter was included since feeding back the foot pressure is the most common approach in the literature, but this information cannot be obtained from the embedded sensors. Importantly, the feedback schemes were evaluated in an ecological scenario, without altering the normal conditions of prosthesis use (e.g., by blocking vision and/or audition). The participant with transfemoral amputation used the device equipped with feedback to walk outside the lab (overground, upstairs, and downstairs) with and without an additional cognitive task.

## Methods

### Embedded feedback design framework

#### Hardware components and communication

**Fig. 1:**
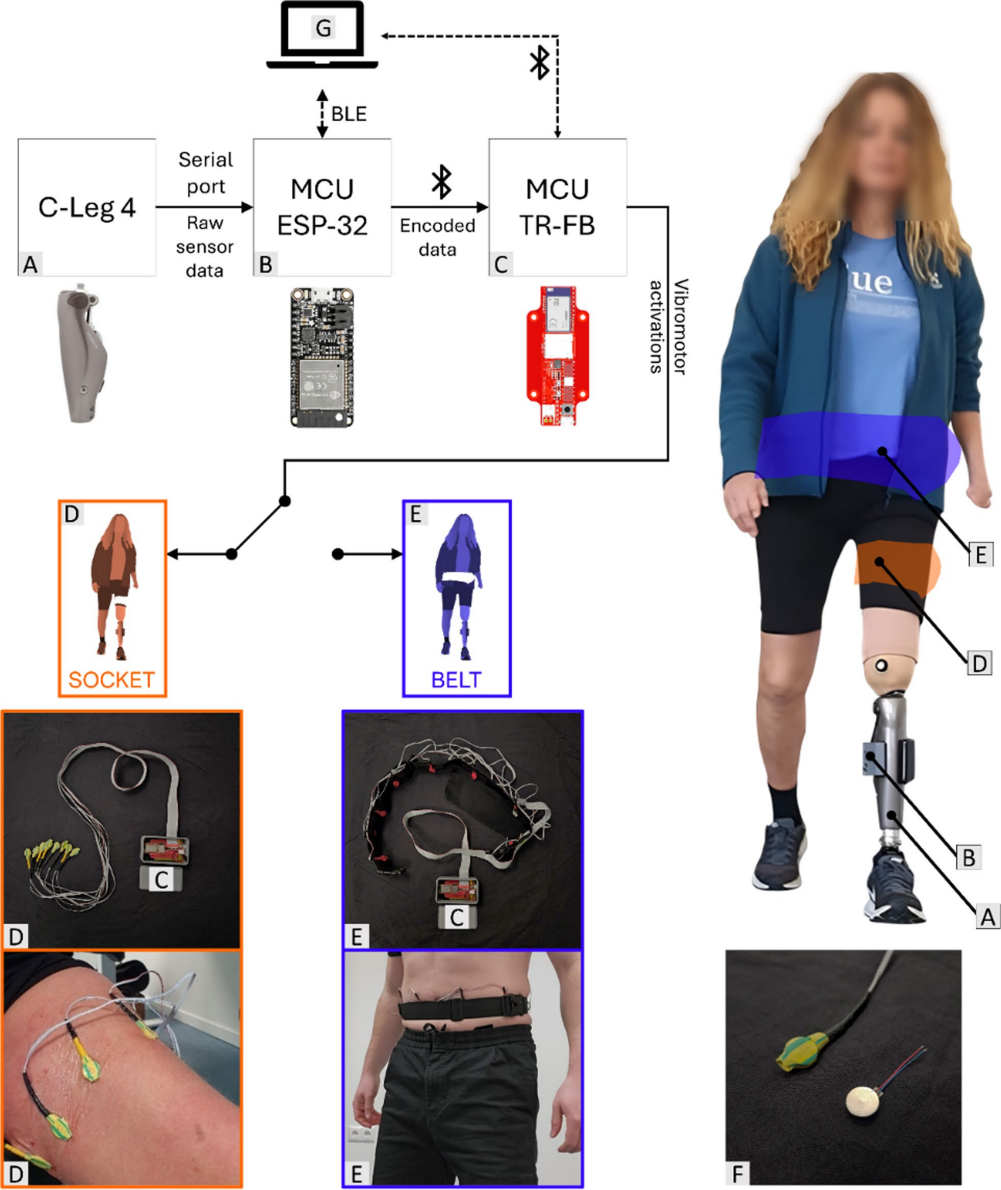
Embedded feedback system–Vibrotactile feedback system using embedded sensors of a microprocessor-controlled prosthesis. (**A**) a C-Leg 4 knee prosthesis (Ottobock SE & Co. KGaA, Duderstadt, Germany), (**B**) an ESP-32 to read the data from the prosthesis (Huzzah32, Adafruit, New York, USA), (**C**) a custom-made vibrotactile device microcontroller (TR-Feedback, Novi Sad, Serbia), (**D**) a brace with 8 vibromotors to place the system around the thigh/residual limb inside the socket (referred to as “Socket” configuration), (**E**) a belt with 8 vibromotors to place the system around the waist (referred to as “Belt” configuration), (**F**) the type of vibromotors used (VC1234B016F, Vybronics, New York, USA), and (**G**) a laptop computer (Intel(R) Core(TM) i7-11800H @ 2.30 GHz, 16 GB RAM) equipped with Bluetooth 5.2)

The framework for the fast prototyping and assessment of the feedback, displayed in Fig. 1, consisted of a custom-made vibrotactile system (TR-Feedback, Novi Sad, Serbia) [60–62] which controlled vibration profiles of 8 vibromotors based on the data acquired from the embedded sensors of a C-Leg 4 knee prosthesis (Ottobock SE & Co. KGaA, Duderstadt, Germany). To fetch the sensor data, an ESP-32 (Huzzah32, Adafruit, New York, USA) was connected to the prosthesis via a serial communication protocol. The ESP-32 retrieved information from the knee angle encoder (knee angle (°) and angular velocity (°/s)), the normalized knee damping values (0–100 activation) for the flexion and extension behavior, gait phases identified by a finite state machine (FSM) and IMU information (orientation and acceleration). The sensor data was sampled and read at 100 Hz.

The ESP-32 module also implemented the feedback encoding that translated the sensor data into stimulation commands, which were then sent to the microcontroller of the TR-Feedback system via Bluetooth connection at 100 Hz. The feedback system activated the motors according to the received commands and the stimulation profiles were delivered to the prosthesis user. The vibromotors were arranged in series, and two configurations were developed to compare their impact on the participants’ perception, where the motors were placed either around the waist or in the socket around the thigh/residual limb, respectively. All vibromotors used eccentric rotating masses (VC1234B016F, Vybronics, New York, USA), operated at a nominal voltage of 3.3 V, with a minimal vibration frequency of 150 Hz, and a force corresponding to the weight of 2 Grms. The motor vibration intensity could be modulated from 0% (no vibration) to 100% (maximal activation). A laptop computer (Intel(R) Core (TM) i7-11800H @ 2.30 GHz, 16 GB RAM) equipped with Bluetooth 5.2 was used during the psychophysical assessment to trigger the vibration profiles directly (direct communication between the laptop and the TR-Feedback system). During the out-of-the-lab assessment, however, the laptop was only employed to switch between the different feedback schemes tested. The experimenter used the laptop to set the stimulation parameters (e.g., sensation thresholds) and select the active encoding scheme, download this information to the ESP-32 via BLE connection, and after that, the feedback system functioned independently.

#### System configurations

**Fig. 2:**
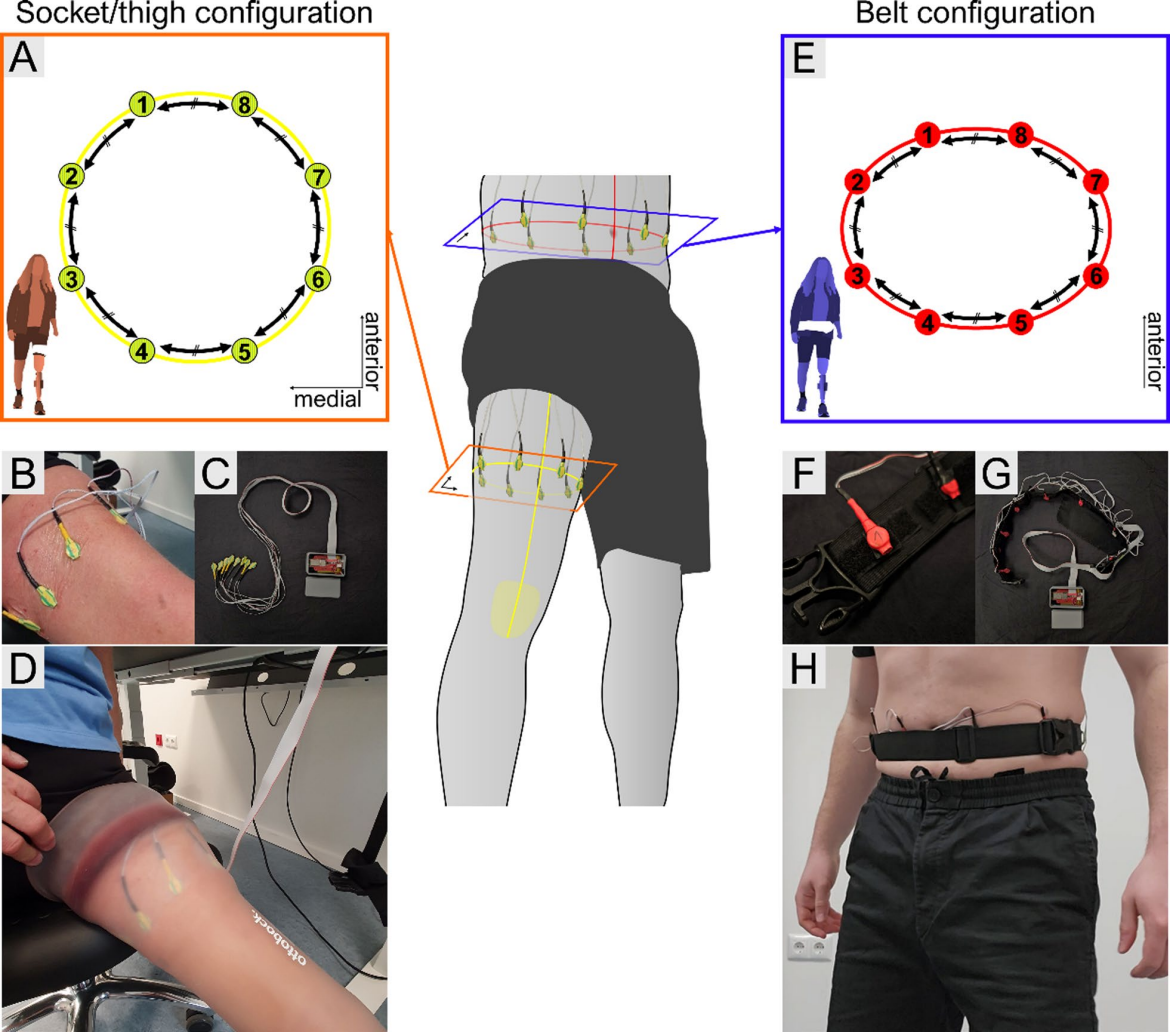
Vibromotor placement–Placement of the vibromotors in the participant with lower-limb amputation and able-bodied participants. The 8 vibromotors were equally spaced either (**A**-**D**) around the thigh/residual limb in the Socket configuration, or (**E**–**H**) around the waist in the Belt configuration, based on the individual thigh/waist circumference

The two configurations of the system placement tested in this study are displayed in Fig. 2. The vibromotors were placed either around the thigh/residual limb or around the waist. In both cases, they were evenly spaced to maximize the distance between 2 adjacent vibromotors, which naturally varied across participants due to differences in thigh and waist circumferences.

When placing the motors around the thigh/residual limb, a point was marked at the mid-length of the thigh along its long axis, using an erasable pen. In able-bodied participants, the point was aligned with the central line of the patella. In the participant with a transfemoral amputation, the point was located on the most anterior part of the curvature of the residual limb. Then, the thigh circumference was measured using a sewing meter and the motors were positioned at equidistant locations around the thigh/residual limb starting from the marked point. The motors were secured using a Tegaderm™ dressing, to avoid further motion. Additionally, for the able-bodied participants, a strap was placed to completely secure the system and increase comfort when wearing a bypass knee. For the participant with a transfemoral amputation, the socket with the prosthesis was placed on top of the vibromotors. Finally, the cables connecting the motors to the feedback controller were routed outside the socket, and the controller of the vibrotactile system was placed inside a fanny pack worn at the waist.

When placing the motors around the waist, they were attached inside a custom-made belt, which allowed moving the vibromotors freely along the belt using Velcro attachments. The microcontroller of the vibrotactile system was placed in a belt pocket. The motors were positioned equidistantly around the waist, taking the navel of the participant as the central landmark.

### Experimental assessment

#### Participants

Ten able-bodied participants AB1-10 (2 females and 8 males with a mean age of 26.8 ± 4.1 years) and one participant with a transfemoral amputation TFA (female, 57 years old) participated in the experiment. Able-bodied participants were eligible if they were at least 1.70 m, which ensured smooth walking with the bypass prosthesis. This constraint did not apply to the participant with lower-limb amputation, as the prosthesis was custom-fitted for her by a certified prosthetist. The participant with a TFA was required to be able to walk independently overground and on stairs for at least 1 min, without the assistance of an external person, and have prior experience using an MP knee.

The characteristics of the able-bodied participants and the participant with TFA are displayed in Table S1 and Table S2 in the Supplementary Materials, respectively. Each participant was provided with an informational brochure about the experiment and signed an informed consent form. The research protocol was approved by the Research Ethics Committee of Region Nordjylland, Denmark (approval number N-20210033).

#### Overview

The goal of this study was to evaluate the perceptual characteristics and functional impact of four vibrotactile feedback schemes integrated into a lower-limb prosthesis. The experimental protocol consisted of two parts: a psychophysical assessment to characterize the perception of vibrotactile stimulation under controlled conditions ("Psychophysical assessment of the Belt and Socket configurations"), and an out-of-the-lab assessment to examine the impact of the feedback during daily walking scenarios ("Preliminary out-of-the-lab assessment of feedback schemes").

All participants (both able-bodied participants and the participant with TFA) completed the psychophysical assessment, which lasted approximately 1.5 to 2 h. This session included amplitude-related tests (sensation threshold (ST) and just-noticeable difference (JND)), as well as a spatial-related test (spatial discrimination (SD)). These assessments were conducted while participants walked on a treadmill: the participant with TFA used her custom-fitted prosthesis, while able-bodied participants used a bypass adapter to walk with the same prosthesis. All participants used both the Belt and Socket feedback configurations.

To explore usability under realistic conditions, the participant with TFA was invited to participate in a separate out-of-the-lab evaluation. This session lasted approximately 2 h, including rest breaks. She experienced and evaluated four feedback modalities (knee angle, hybrid, damping, and force feedback, as described in section "Preliminary out-of-the-lab assessment of feedback schemes"). Walking tasks included overground and stair ambulation, with and without a concurrent cognitive task. In addition to biomechanical gait analysis, we assessed cognitive load, perceived usefulness, and user preferences for each feedback type. Timelines for both experimental parts are shown in Fig. 4 and Fig. 9.

#### Psychophysical assessment of the Belt and Socket configurations

##### Experimental setup

During the psychophysical evaluation, the able-bodied participants walked with the C-Leg 4 by using a custom-made bypass knee system, thereby partially replicating the conditions of walking with a prosthesis.

**Fig. 3:**
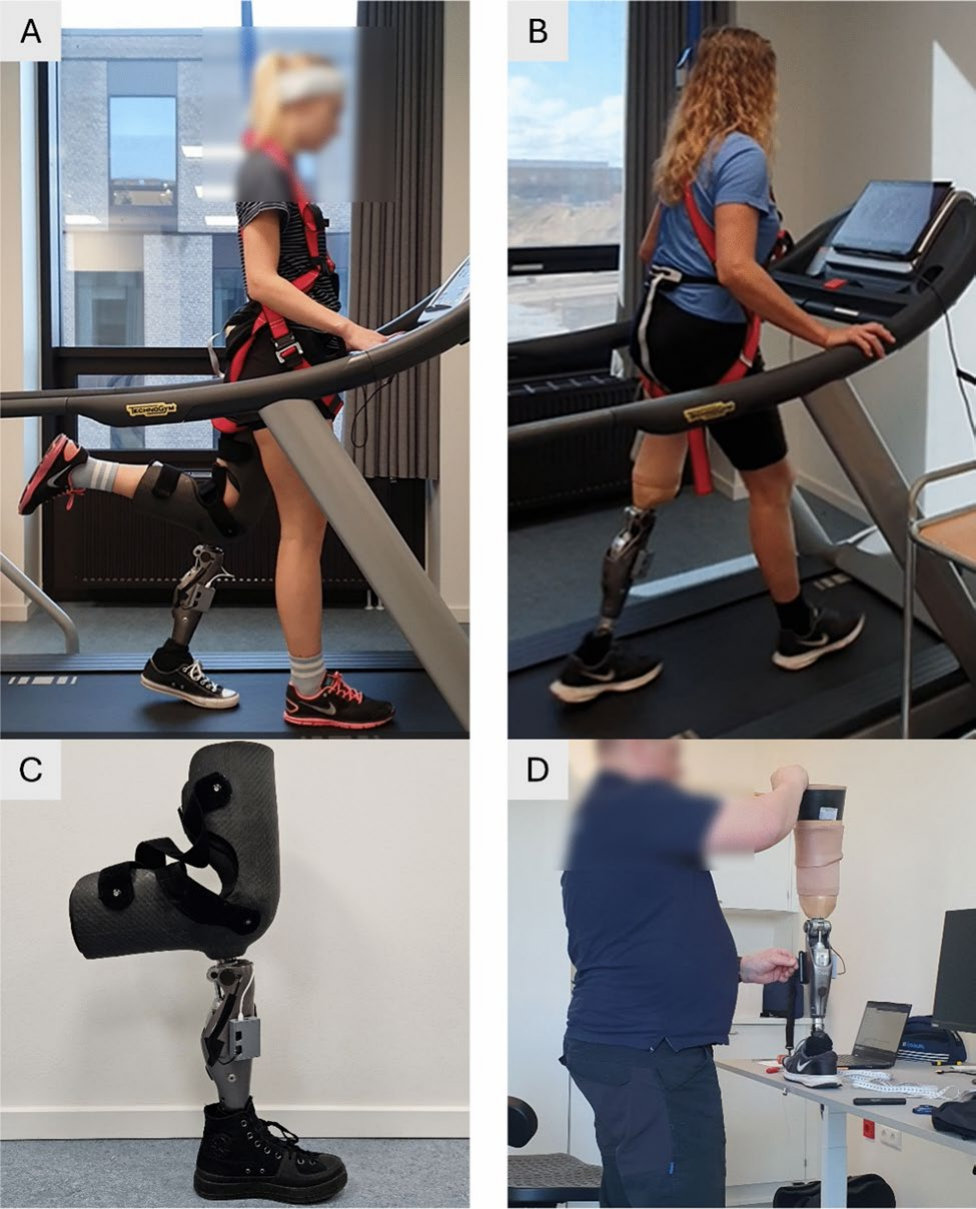
Experimental setup–The setup for the psychophysical assessment in (**A**) able-bodied participants and (**B**) the participant with TFA. Able-bodied participants wore a bypass knee (**C**) whereas a prosthetist (**D**) attached the C-Leg prosthesis to the TFA’s own socket and then aligned the system according to the standard clinical practice

To this aim, the able-bodied participants placed their knee inside a carbon-fiber cast to which the prosthesis was attached as displayed in Fig. 3 (A and C). The comfortable and secure placement of the bypass system was ensured by additional foam patches that could be added/removed, and straps that kept the leg in place. Several prosthesis parameters, such as the body weight and prosthetic knee axis height were adjusted in the C-Soft Plus software, and a standard calibration was performed following the recommendation of the prosthesis manufacturer to adapt the prosthesis to the participant and ensure stable behavior during gait. More specifically, the calibration procedure required the user to stand still with the knee extended within the operating range, while the C-Soft Plus software automatically updated the internal controller parameters of the C-Leg to align sensor readings and mechanical characteristics. The details of these internal adjustments are proprietary to the prosthesis manufacturer.

The participant with TFA wore the same C-Leg 4 prosthesis (Fig. 3–B) as the able-bodied participants. A certified prosthetist (Ortos Bandagist Klinik, Aalborg, Denmark) adjusted the height of the prosthesis using a standard pylon, ensured proper static and dynamic alignment of the knee and ankle joints, and fine-tuned the system for the participant using C-Soft Plus (Fig. 3–D). The parameters were fine-tuned during overground walking but also stair ascent and descent to properly adjust the damping of the knee joint in those scenarios, following the standard practice. As indicated in Table S2, the participant’s usual prosthesis is a Rheo Knee XC. The C-Leg 4 was used only for this study, and a 15-min familiarization period with the new device was provided following the fitting. Importantly, both systems are of the same general type (an MP prosthesis).

##### Experimental protocol

**Fig. 4:**
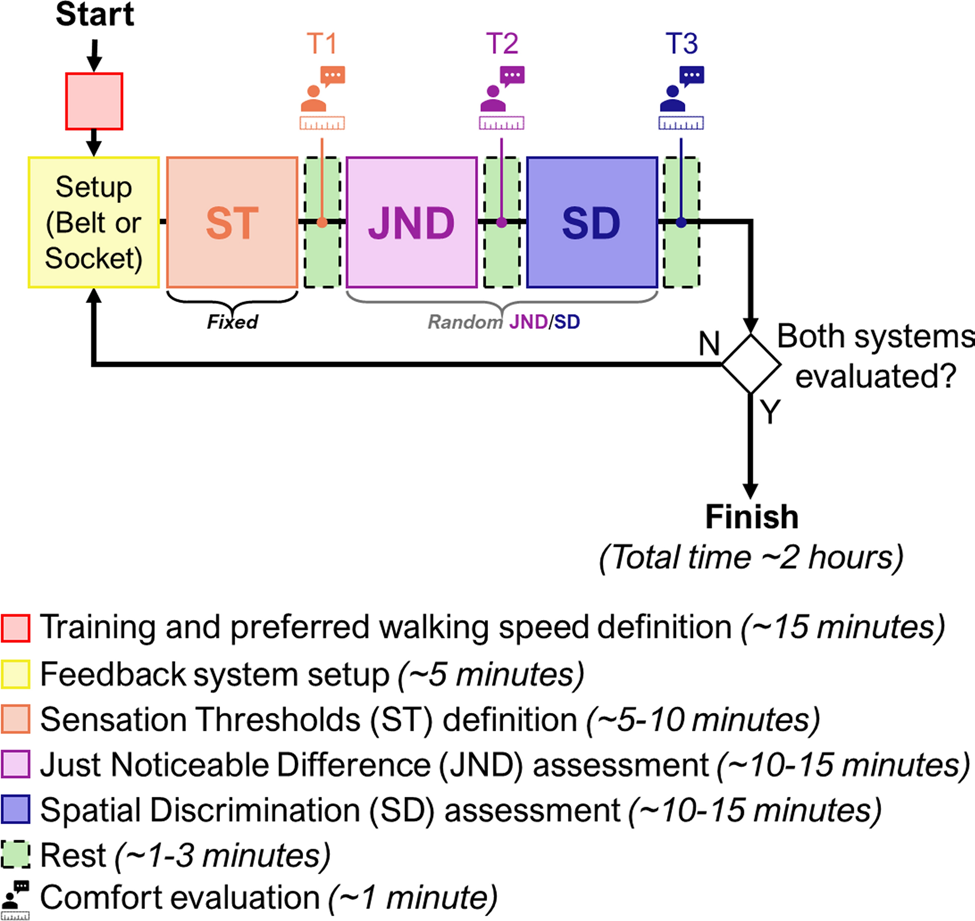
Experimental protocol–After mounting the prosthesis, the participants briefly practiced walking on a treadmill at their preferred walking speed. Then, we placed the vibrotactile system in one of the configurations (randomly chosen between Belt and Socket configuration) and followed with the psychophysical assessment at the preferred walking speed. We first determined the Sensation Thresholds (ST), followed by the Just Noticeable Difference (JND) and the Spatial Discrimination (SD) tests in random order. We evaluated the subjective experience of comfort at three moments during the experiment: “T1”, right after assessing the sensation thresholds, “T2”, after the second evaluation (JND or spatial discrimination), and “T3” after the last evaluation. After one configuration was evaluated, the system was mounted in the other configuration and assessed following the same protocol

The psychophysical assessment aimed to compare the differences in comfort, amplitude, and spatial perception between the Belt and Socket configurations. In addition, test conditions were selected so that they reflect the vibrotactile patterns used later during functional assessment of feedback (see section "Preliminary out-of-the-lab assessment of feedback schemes"). The session duration was approximately 2 h, and this included the setup of the device, the psychophysical evaluations (amplitude and spatial assessments), the comfort assessment, and regular breaks in between as shown in Fig. 4.

The able-bodied participants were first trained for approximately 10 min to learn how to walk with the prosthesis on the treadmill. The prosthesis was set up as described earlier and the participants were invited to stand up on the treadmill and hold the treadmill’s lateral bars. A safety harness was then placed to catch the participant in case of a fall. Nevertheless, the participants were told to hold the bars during the experiment whenever they felt the need to do so. They started walking at a slow pace (0.14 m/s) and practiced how to trigger the swing phase of the prosthesis at each gait cycle, which is a critical transition to have a quasi-natural gait behavior. All participants learned without major difficulties how to perform this task and therefore walk with the device.

The next step was to define a preferred walking speed, which was then kept constant during all the assessments. The treadmill was started at 0.14 m/s and the speed was increased in increments of 0.028 m/s. The participants were asked to indicate when a walking speed that felt comfortable and could be sustained for the entire experiment was reached. Then, the speed was increased by 0.14 m/s and gradually reduced in decrements of 0.028 m/s, and again, the participants indicated a comfortable speed. We then computed the average of these two values and adopted this as the preferred walking speed.

Once we determined the preferred walking speed, the feedback system was set up using the Belt or Socket configuration following a pseudorandom ordering across participants, and the psychophysical tests were performed as shown in Fig. 4. The amplitude assessment included the measurement of the sensation threshold (ST) and just noticeable difference (JND) to characterize the sensitivity and resolution in perceiving the intensity modulation afforded by each configuration, respectively. Similarly, the aim of the spatial discrimination (SD) assessment was to determine how well the participants could recognize specific patterns of vibromotor activations (stimulation location) when using the two feedback configurations. The ST assessment was always the first test to be performed as the results of this test were required to perform the other assessments. The order of the JND and SD was assigned pseudorandomly across participants. Finally, the order of sub-conditions in the ST, JND, and SD (described respectively in the following sections) was also pseudorandom. Importantly, all tests were conducted while the participants walked on the treadmill since our previous study showed that walking might alter the perception of stimulation [33]. We predefined the order of conditions for all able-bodied participants before the recruitment (pseudorandomization) to ensure that the sequence of conditions was indeed different across participants.

The participants rested for approximately 3 min between each assessment, around 1 min between sub-conditions, and for about 10 min when we changed the feedback configuration (from Socket to Belt or vice versa). During the breaks between the ST, JND, and SD, the comfort was evaluated, by asking the following question to the participants: “How much would you rate the comfort of the feedback system? Rate from 0 to 10 (0.5 increments)”.

##### Amplitude assessment

*Sensation thresholds (ST) assessment*

**Fig. 5:**
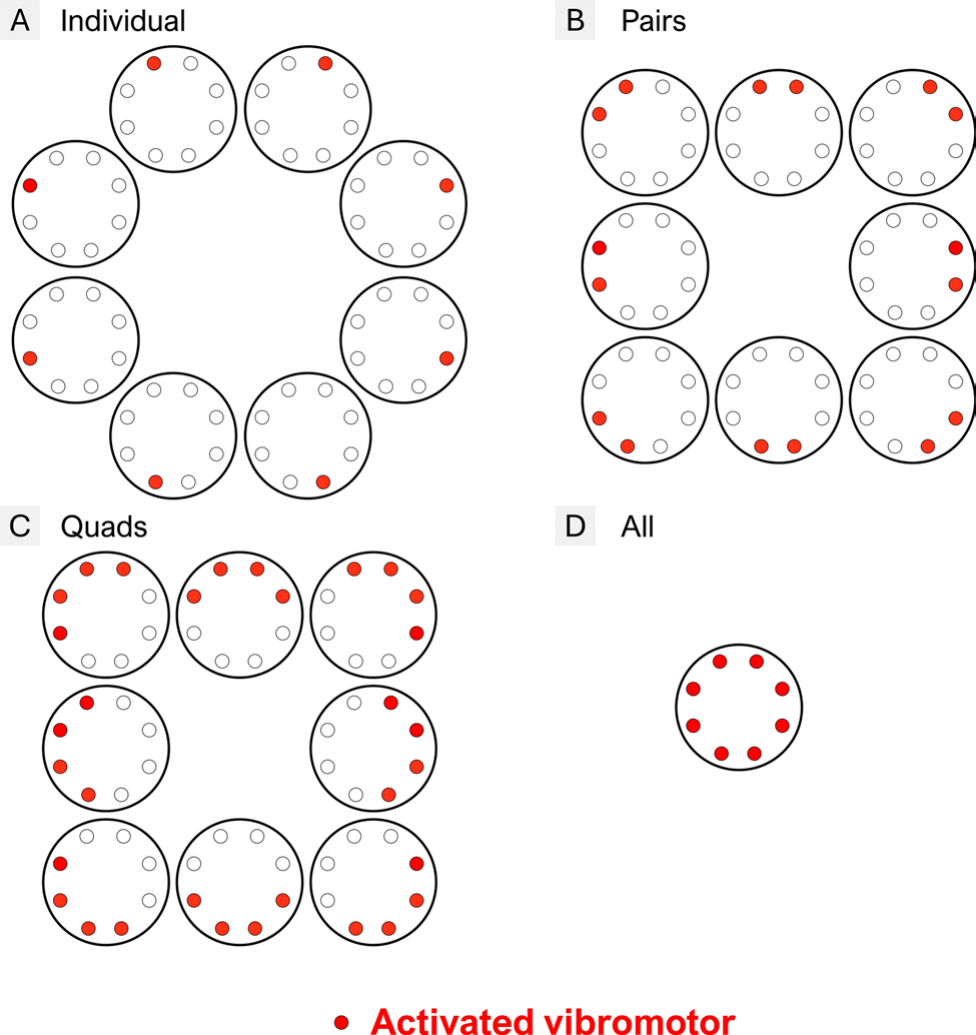
Sensation Thresholds (ST) assessment–The sensation thresholds (ST) were assessed by activating different numbers of motors (sub-conditions of ST). The ST was determined for (**A**) each motor individually and for (**B**) pairs, (**C**) quads, and (**D**) all vibromotors. The order was pseudorandom across the four sub-conditions, and the ST was computed as the average ST across all combinations of motor activations in each sub-condition (8 for 1, 2, and 4 motors and 1 for 8 motors)

We assessed the ST in four sub-conditions, namely, when activating individual vibromotors (“Individual”–Fig. 5–A), pairs of vibromotors (“Pairs”–Fig. 5–B), quads of vibromotors (“Quads”–Fig. 5–C), and all vibromotors at once (“All”–Fig. 5–D). This was done to investigate how the spread of the vibration impacts the sensation thresholds in the two feedback configurations. To evaluate the sensation thresholds, we used the ascending method of limits [63]. The vibromotors comprising the pattern that was tested were activated at 1% of the maximal activation and the intensity was then progressively increased in steps of 1% until the participant indicated that they perceived the stimulation. This evaluation was performed for each combination of 1, 2, and 4 motors (8 for each) and once for all 8 motors, as shown in Fig. 5. The final ST for each number of motors was calculated as the average across tested combinations. The participants walked on the treadmill at the preferred walking speed and each stimulation was triggered randomly during the gait cycle and lasted for 2 s. This duration, which is also used for the following tests, was intentionally chosen based on prior evidence suggesting that tactile sensitivity may vary across different phases of the gait cycle when delivered to the limb [64], but not at the waist [65]. By extending the stimulus across multiple steps or gait phases, we ensured that participants would reliably perceive the stimulation regardless of when it occurred.

*Just noticeable difference (JND) assessment*

The JND represents the minimum change of vibration intensity that can be perceived by the participants. Although assessing JND with a single vibromotor might offer finer sensitivity to small amplitude changes, we opted to use simultaneous activation of all eight vibromotors to reflect the configuration used in the knee angle feedback modality (see section "Preliminary out-of-the-lab assessment of feedback schemes"). This approach ensured that the measured perceptual sensitivity was directly relevant to the type of amplitude modulation applied in the actual feedback, thereby improving the ecological validity of the assessment. The “weighted” up/down method was used in this assessment, targeting the JND that corresponds to 66.7% of correct detections [59]. The participant was presented with two consecutive stimuli in a random order, namely, the baseline and test stimulus. The baseline was always constant and set to ${I}_{baseline}=round(ST+0.15\times\left(100-ST\right))$, while the initial intensity of the test stimulus was set to ${I}_{test}=round(ST+0.9\times\left(100-ST\right))$, where ST is the sensation threshold in percent. Therefore, the intensity of the test stimulus (${I}_{test}$) was higher than the baseline and changed adaptively, based on the participant’s responses (staircase procedure). More specifically, when the participants guessed correctly, the difference between the baseline and test stimulus intensities was reduced by decreasing the value of ${I}_{test}$; conversely, if the participant responded incorrectly, ${I}_{test}$ was increased. Therefore, the test stimulus converged toward the participant’s discrimination threshold.

To ensure faster convergence during initial guesses, the amplitude change was initially set to 5% of the maximum vibromotor intensity (100%). This coarse step size was maintained until the relative difference between the baseline and test amplitude was below 20% or the participant gave an incorrect response. After that, the procedure switched to a finer staircase, with the test intensity (${I}_{test}$) decreasing by 1% after correct responses and increasing by 2% after incorrect ones.

The staircase procedure was finished after 10 reversals were reached, where a reversal was defined as a change of the test stimulus intensity from increasing to decreasing or vice-versa. The JND value corresponded to the average intensity of the last 5 reversals. The staircase also finished if the participant correctly guessed the test stimulus 7 consecutive times at the smallest amplitude difference (1%), and in this case, the JND was set to 1%. Each vibration provided to the user lasted for 2 s, with a pause of 1 s in between the two stimuli. The Weber fraction was then computed as the ratio of the JND and the baseline intensity.

##### Spatial assessment

*Spatial discrimination (SD) assessment*

**Fig. 6:**
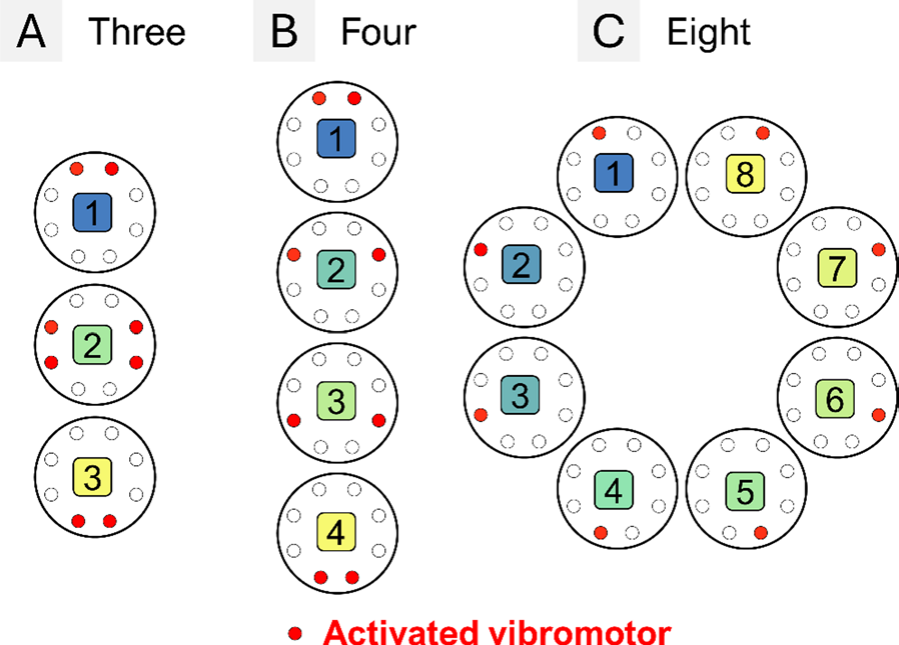
Spatial discrimination (SD) assessment–The participants were asked to recognize different numbers of spatial stimulation patterns (sub-conditions) while walking on a treadmill with the feedback in the Belt and Socket configuration. The tasks included discriminating (**A**) 3, (**B**) 4, and (**C**) 8 stimulation patterns. C was the most difficult task while A and B were used for the feedback encoding during the online assessment (see section "Preliminary out-of-the-lab assessment of feedback schemes").

In this test, the participants were asked to recognize a delivered spatial stimulation pattern chosen randomly from a set of possible profiles. The test comprised three sub-conditions, one representing the most difficult perception task, in which the participant was asked to identify each of the 8 individual vibromotors locations (see Fig. 6–C), and two sub-conditions with fewer options to recognize (see Fig. 6–A and B). These two sub-conditions were specifically designed to include the spatial patterns used for feedback encoding during the online evaluation (see hybrid and damping feedback in section "Preliminary out-of-the-lab assessment of feedback schemes"), thereby ensuring continuity between the psychophysical assessment and applied feedback schemes. Also, the selection of these patterns was dictated by the need to encode discrete feedback states while maximizing the distinctiveness and clarity of vibrations associated with each state. All the tests were performed at the maximal amplitude (100% of activation of the vibromotors), and each vibration provided to the participant lasted for 2 s. The schematic representations of the patterns to be recognized were placed as a reminder in front of the participants while they walked on the treadmill.

Each sub-condition consisted of 3 parts: a familiarization phase, a reinforced learning phase, and an evaluation. During familiarization, the different stimulation patterns were delivered to the participants 2 times in a random order. The experimenter indicated verbally the pattern that was presented and showed it on the figures. In the reinforced learning phase, the participants were presented with the stimulation patterns in a random order and asked to guess the presented pattern. The experimenter then indicated if the participant was correct and when they were wrong, showed them the correct pattern. Each pattern was delivered 5 times. The evaluation phase was identical to the reinforced learning, but each pattern was presented 10 times and the correct pattern was not disclosed to the participant. A 1-min break was introduced between each phase, and a 2 to 3-min break was inserted between each sub-condition. During the evaluation phase of the task with the eight patterns, there was an additional break after the 40th stimulus to avoid mental fatigue. The order of sub-conditions was pseudorandomized across participants.

##### Outcome measures and statistics

The results of the able-bodied participants were analyzed statistically by conducting repeated measures two-way ANOVA with the factors of feedback configuration (two levels: “Belt” and “Socket”) and the number of active motors (four levels: “Individual”, “Pairs”, “Quads”, and “All”) for ST, feedback configuration and the number of spatial patterns (three levels: “Three”, “Four” and “Eight” choices) for SD and feedback configuration and time instants of the comfort assessments (three levels: T1, T2, and T3, as shown in Fig. 4). Before conducting the ANOVA, the Shapiro–Wilk test was conducted to assess the normality of the dependent variable within each group (p > 0.05). Then, Mauchly's test of sphericity was conducted to assess the assumption of sphericity for the repeated measures factors. Levene's test was conducted to assess the homogeneity of variances for the dependent variable across the different groups (p > 0.05). In case ANOVA revealed significant differences, post-hoc pairwise comparisons were conducted with Bonferroni corrections. A paired t-test was applied to compare WF between the two feedback configurations. The threshold for statistical significance was set to p < 0.05. The statistical analysis was performed in SPSS 28 (IBM, Armonk, NY, USA).

#### Preliminary out-of-the-lab assessment of feedback schemes

##### Encoding schemes

**Fig. 7:**
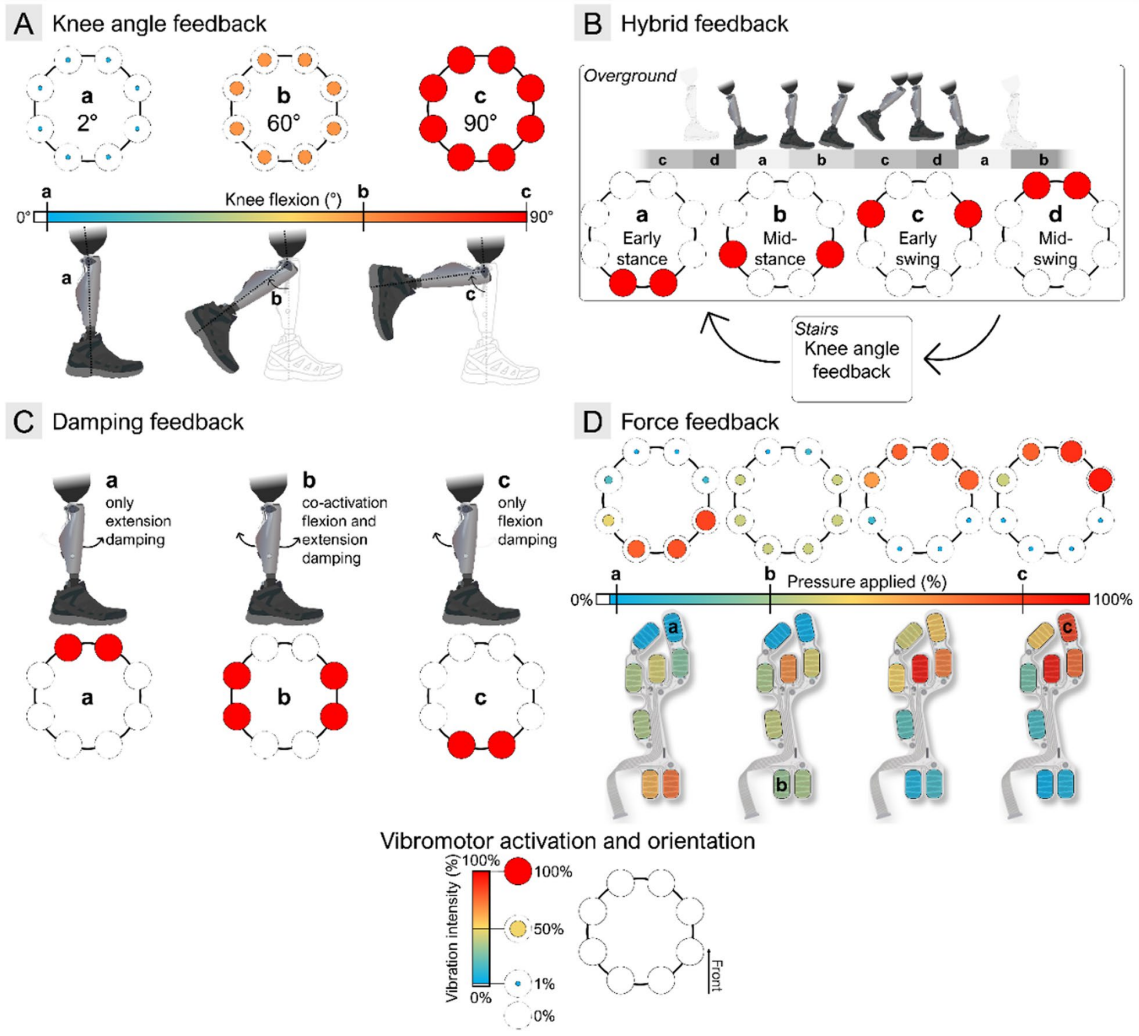
Feedback encoding** –** The figure displays the (**A**) knee angle, (**B**) hybrid, (**C**) damping, and (**D**) force feedback encoding schemes tested during our out-of-the-lab assessment. Over all the panels, the color grading and size of the circles indicate the percentage of vibration intensity of each vibromotor, from low intensity (small blue circle), to mid (average yellow circle), to high intensity (big red circle). For the knee angle feedback (**A**), the continuous change of knee angle is mapped onto the vibration intensity of all 8 vibromotors simultaneously. Three examples of vibrations are shown for angles of (**a**) 2°, (**b**) 60° and (**c**) 90°. For the hybrid feedback (**B**), the feedback alternates between the overground mode, in which gait phase information, namely, (**a**) the early stance, (**b**) mid-stance, (**c**) early swing, and (**d**) mid-swing is given to the user; and the stairs mode, in which the knee angle feedback is provided as in (**A**). For the damping feedback (**C**), the information about the high knee damping in (**a**) extension, (**b**) flexion and extension, or (**c**) flexion, is given to the user with the three patterns displayed. Finally, for the force feedback (**D**), a continuous mapping of the force below the sole is projected onto 8 vibromotors, according to the algorithm described in depth in [59]. We show here 4 pressure profiles, with the pressure applied (expressed in percentage of maximal pressure defined during calibration), at different FSRs on the insole (**a**, **b**, **c**)

In this part of the experiment, we tested the knee angle feedback (Fig. 7–A), hybrid knee angle and gait phases feedback (Fig. 7–B), the damping feedback (Fig. 7–C), and the force feedback (Fig. 7–D) in the participant with TFA.

The knee angle (Fig. 7–A) information was conveyed by linearly mapping the angle to the intensity of 8 simultaneously activated vibromotors. This type of feedback is commonly used in the literature [14–20, 48, 52]. To calibrate the feedback, we first recorded the gait of the participant with TFA during a 2-min walking test involving overground, upstairs, and downstairs walking. The minimal and maximal knee angle amplitude achieved during this test was determined, and the mapping from the angle into the vibration amplitude was defined as:

1 $$ \left\{ \begin{aligned} & VIB_{all} = 0\% \,\,\,\,\,\,\,\,\,\,\,\,\,\,\,\,\,\,\,\,\,\,\,\,\,\,\,\,\,\,\,\,\,\,\,\,\,\,\,\,\,\,\,\,\,,for\,\alpha <_{\min } or\,\alpha > 95^{ \circ } \\ & VIB_{all} = \frac{{(\alpha - \alpha_{\min } ) \times (100 - ST)}}{{\alpha_{{{\mathrm{max}}}} - \alpha_{{{\mathrm{max}}}} }}\,\,\,,for\,\alpha_{\min } \le \alpha \le \alpha_{{{\mathrm{max}}}} \\ & VIB_{all} = 100\% \,\,\,\,\,\,\,\,\,\,\,\,\,\,\,\,\,\,\,\,\,\,\,\,\,\,\,\,\,\,\,\,\,\,\,\,\,\,\,,for\,\alpha > \alpha_{{{\mathrm{max}}}} \\ \end{aligned} \right. $$

where $VI{B}_{all}$ is the vibration amplitude of all the vibromotors in percent of maximum intensity, ST the percent sensation threshold, $\alpha$ the current knee angle value as measured by the encoder, ${\alpha }_{min}$ the minimum knee angle recorded during the walking trial plus 0.5°, and ${\alpha }_{max}$ the maximum knee angle recorded during the walking trial. The first condition described in Eq. (1), was defined to allow the participants to switch off the vibration when resting by fully extending or fully flexing the knee. The periodical flexion and extension of the knee during overground walking and stair descent leads to pulsating vibrotactile sensation, as the intensity increases and decreases in sync with the knee angle. During stair ascent, however, the range of intensity modulations depends on how much the participant can bend the knee (e.g., some users climb stairs with straight knees and in this case, no stimulation would be provided).

The hybrid feedback (Fig. 7–B) consisted of a combination of knee angle feedback (used during stair ascent and descent) as described above (Fig. 7–A), and the feedback of gait phases during overground walking. The latter approach is also commonly used in the literature [18–20, 43, 50]. The gait phase feedback indicated 4 different phases of the gait cycle, namely, early stance, mid-stance, early swing, and mid-swing, estimated by the C-Leg 4 proprietary software during overground walking. Therefore, while walking overground, the feedback delivers four vibrotactile patterns from the posterior to the anterior side of the residual limb, as the gait phases sequentially progress from early stance to mid-swing, as shown in Fig. 7–B. However, the gait phases are identified only during overground walking, and hence, when another modality was detected by the leg (e.g., ambulating over stairs or slopes), the feedback switched to conveying the knee angle, using the encoding scheme described earlier.

The damping feedback (Fig. 7–C) is a novel method that has not been tested in the literature. The C-Leg 4 (Ottobock SE & Co. KGaA, Duderstadt, Germany) attempts to replicate the function of a biological leg by modulating two internal damping parameters (one for flexion and one for extension) by adjusting the hydraulic valves actuated by internal servo motors [29]. These parameters vary according to the proprietary control logic and, for the purpose of this feedback, were normalized between 0% (no damping) and 100% (maximum damping). In principle, the feedback could have provided the damping values directly and continuously (e.g., vibration intensity proportional to the instantaneous damping); however, such encoding could be difficult to interpret because these signals vary rapidly, as we have seen in the pilot tests. Therefore, we have implemented a simplified threshold-based approach to clearly indicate to the user the periods of high and low, velocity-dependent resistance, rather than continuously changing values. This design provided interpretable, functionally meaningful cues without requiring extensive user training. Contrary to the conventional approaches that transmit the sensory consequences of the movement (gait kinematics or dynamics), this feedback method conveyed information about the internal state of the prosthesis itself. Three spatial patterns of vibrations were delivered to the participant to indicate that the knee damping has been set by the prosthesis controller to be above 50% in flexion (posterior vibration), extension (anterior vibration) or in both directions (medio/lateral vibration). The 50% threshold was selected empirically during pilot testing to indicate periods of perceptibly elevated damping relevant for weight acceptance during stance and limb motion during swing. Both flexion and extension damping are increased before initial contact during overground walking and stair climbing (provided these phases are correctly recognized by the internal prosthesis controller). In response, the participant receives the mediolateral vibration pattern, indicating that the prosthesis is ready to receive the body weight without giving away. The cessation of this cue conveys to the user that the knee is ready to flex, for example, during the later phase of stair descent, prompting them to exercise caution. During the swing phase of normal walking, the prosthesis increases extension damping (early swing) and then flexion damping (late swing), which activates anterior and posterior stimulation. Therefore, the feedback informs the user of the joint state in real-time, supporting safer weight transfer onto the prosthetic side.

Finally, a force feedback scheme (“OmniFeel”, Fig. 7–D) presented in our recent publication [59], has been also included in the test. This feedback provided direct information about the pressure profile under the foot sole in an intuitive manner thanks to a sensorized insole (ActiSense, IEEE, Luxembourg) and omnidirectional encoding using a vibromotor array. During overground walking, the pressure normally progresses from heel to toe and in response, the vibration sequence moves smoothly from the posterior to the anterior aspect of the residual limb. In stair ascent and descent, vibrotactile sensation indicates the part of the foot in contact with the stair. For instance, a posterior and lateral vibration conveys to the user that the heel and midfoot are properly placed and that they can safely make a step (without the need to check this visually).

##### Experimental setup and protocol

**Fig. 8:**
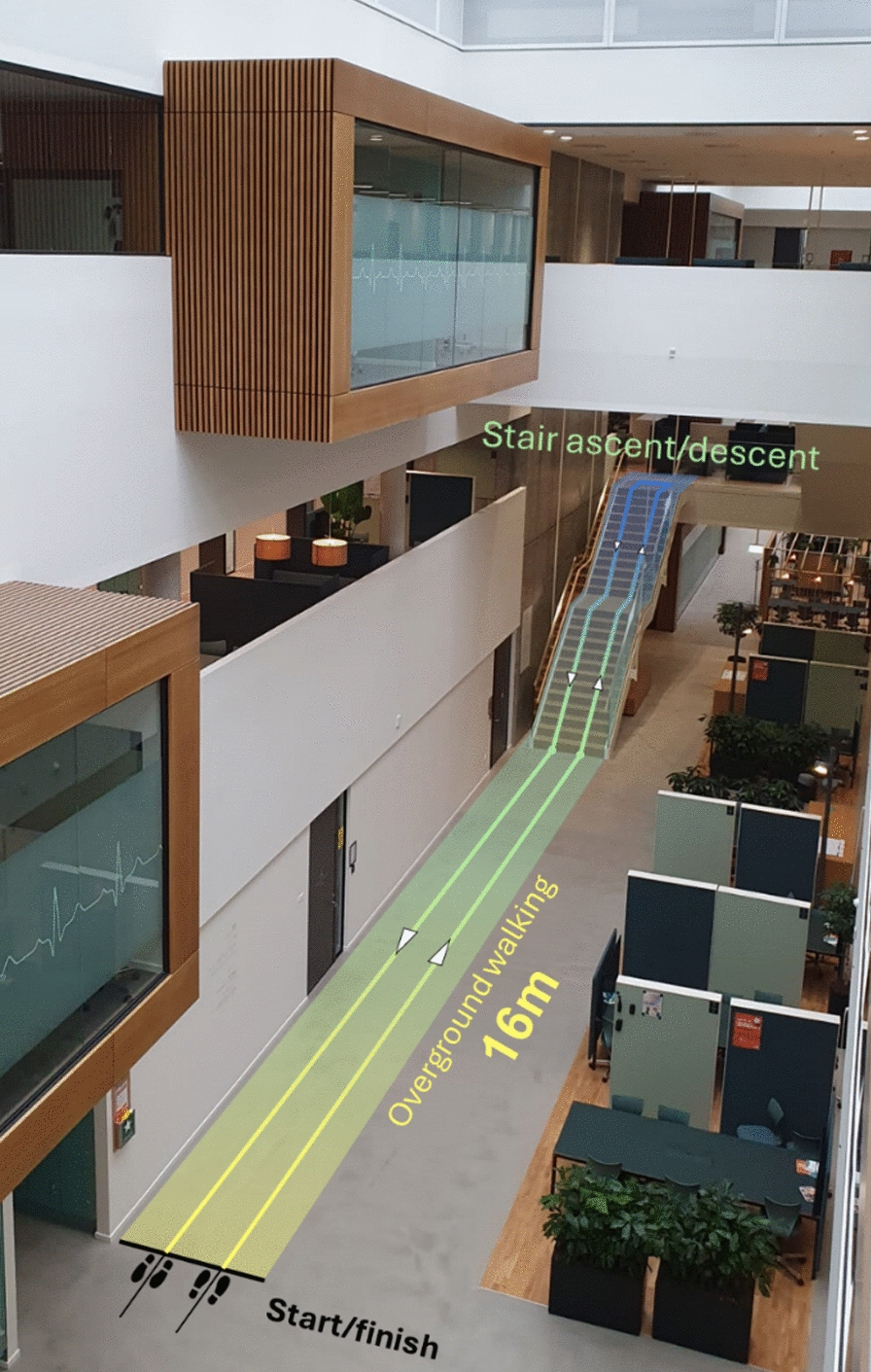
Walking course–The participant with TFA walked over the displayed walking course in the university building. The course consisted of a total of 32 m of overground walking and a staircase with 25 stairs. The participant walked over the course, starting and coming back to the same initial position, two times in each feedback condition (4 feedback schemes and no feedback), with and without a concurrent cognitive task (counting backwards in 7s). The gait kinematics was recorded using an MTw Awinda XSens system

Only the participant with TFA participated in this part of the experiment, wearing the feedback system in the Socket configuration. The aim was to perform a preliminary assessment of the impact of multiple feedback schemes from functional, cognitive, and user experience perspectives. The session lasted approximately 2 h and comprised the setup of the feedback device, the functional evaluations, questionnaires, and breaks. Four feedback schemes, described in the previous subsection, were tested while she walked through the university building (Fig. 8). She walked over the flat walkway (16 m), climbed a staircase made of 25 stairs, turned around, and came back to the starting position. With each feedback scheme (as well as with feedback deactivated), she traversed the walking course twice: with and without a parallel cognitive task. The mental task consisted of counting backward in 7s, starting from a random number between 200 and 250. This kind of task is commonly used in dual-task gait assessment and is shown to produce high levels of interference during walking [66–68].

An IMU-based motion capture system (MTw Awinda XSens, Movella, Enschede, Netherlands) was used to evaluate the impact of the feedback schemes, by measuring the spatiotemporal parameters and kinematics of the gait when the participant with amputation performed the walking course. Externally placed IMUs were therefore used only for assessment and not for online control and/or feedback. Eight IMUs were placed in the “Lower body with Sternum” configuration as recommended in the system user manual to track the motion of the ankle, knee, hip, and spine. This configuration was previously used to record and analyze gait kinematics in people with lower-limb amputation [69]. Specifically, the IMUs were positioned on the feet, shanks, thighs, pelvis, and sternum, and the data were recorded at 100 Hz using MVN 2022.0.2 (XSens, Movella, Enschede, Netherlands). We measured the dimensions of the different body parts of the participant with TFA as described in the user manual, and this included the height, foot length, ankle, knee, hip, and shoulder heights, as well as hip and shoulder widths. These parameters were used to customize the general kinematic model to the specific participant [70]. We then performed a calibration, which required the participant to stand still with arms along the body (in “N-pose”), walk a few meters, and return to the initial starting position. Finally, when recording the data, we selected the “multi-level” mode in the software [70], which assumes that the height will vary during walking. To analyze the biomechanical data, we used the same approach as in our previous work [59]. Specifically, for each recording, we: (1) segmented the data into different sections corresponding to overground walking, stair ascent or descent, respectively; after that, we (2) performed post-processing using the Motion Cloud service in MVN Processing software; and then, (3) processed the exported data in MATLAB using the Movella SDK and custom-made scripts to compute key metrics and average them across gait cycles, namely spatiotemporal parameters (stance time, stance percentage, gait cycle time) and joint kinematics (ankle, knee, hip angles, pelvic tilt). The symmetry of those parameters during overground, upstairs, and downstairs walking was computed using the Symmetry Index (SI), expressed on a normalized scale from 0 (complete asymmetry) to 100% (desired symmetry, similar to able-bodied participants) [71].

Once the systems were placed, the participant was introduced to the task. The experimenter performed the walking course alongside the participant, for her to get familiarized with the task and feedback schemes. Each feedback scheme was carefully explained to the participant and then demonstrated while walking for around 5 min with the feedback activated. She was asked to “go as fast as possible, without putting herself in danger” during the whole experiment, and that safety was the most important concern. Also, the experimenter constantly followed the participant to react if necessary (e.g., trips and falls) but this was not required.

**Fig. 9:**
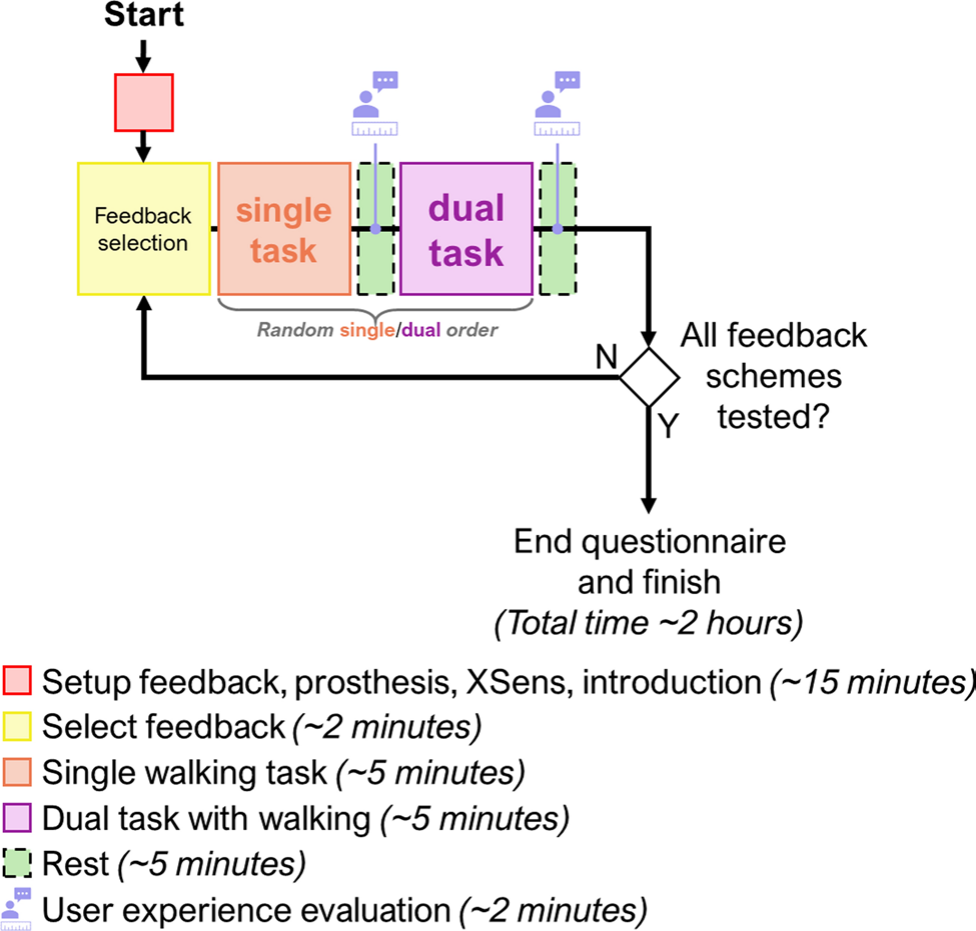
Experimental protocol–After placing the vibrotactile system in the Socket configuration, mounting the prosthesis and the XSens systems, the participant was introduced to the different feedback schemes and the dual task to perform while walking on the walking course. Then, the participant performed the single and dual-task walking in a random order, sequentially with all feedback schemes as well as without feedback. The participant always started and finished the evaluation with a “no feedback” condition and performed the other conditions in a random order. We evaluated the cognitive load after both tasks (single and dual) and in each feedback condition. After all the feedback approaches were evaluated, the participant was asked to respond to the questionnaires that included the ranking of the perceived usefulness of the tested feedback schemes, and rating of the feedback variables)

After this introduction, the main experiment started (see protocol in Fig. 9). The order of the testing of the four feedback schemes was randomly assigned, and no feedback was performed twice (pre- and post-). The condition without feedback (no-feedback) was performed both at the beginning and at the end of the session to account for potential variability associated with task familiarization or fatigue. The four feedback conditions were each tested once to avoid overloading the participant while still maximizing the number of conditions tested within a single session. The participant then walked over the walkway with the selected feedback scheme with or without parallel cognitive task (random order) and rated her perception of the cognitive load after each task. To this aim, we asked the participant to reply to the following question “How cognitively demanding was the task? Rate from 1 to 9” [72]. Using a standard questionnaire, such as NASA-TLX [73], could offer a more comprehensive assessment of the cognitive load. However, its length may increase participant’s fatigue and cognitive burden, whereas the simpler cognitive rating used in this study is practical and requires minimal effort to complete [30]. After that, the participant was presented with another feedback scheme until all schemes were tested. In the end, she was asked to rank the perceived usefulness of the feedback schemes during overground, upstairs, and downstairs walking. The participant was allowed to rank two feedback schemes equally. We also asked the participant to rate various feedback variables according to their presumed usefulness. More specifically, we asked the participant to complete the sentence “I would feel more confident if I had information about […]” and the participant rated each feedback variable from 0 (not needed/would not make me feel more confident) to 10 (totally needed/would definitely make me more confident). The full list of variables included those tested during the present experiment plus extra options to obtain a comprehensive insight into the participant’s preferences, i.e., the angle, angular velocity, and torque of the knee flexion/extension, ankle plantar/dorsiflexion, and ankle inversion/eversion, the orientation of the shank, axial load, force profile below the foot, gait phases, and peak knee angle flexion/extension. The experimenter explained the nature of each of the variables using also the participant’s experience of the online feedback during the session. The participant was told that she could propose other feedback variables, outside those listed in the questionnaire.

## Results

### Psychophysical assessment of the Belt and Socket configurations

The average self-selected walking speed for able-bodied participants was 0.53 ± 0.06 m/s (see individual values in Table 1 in Supplementary Materials). Interestingly, the TFA participant selected a notably higher speed of 0.75 m/s.

**Fig. 10:**
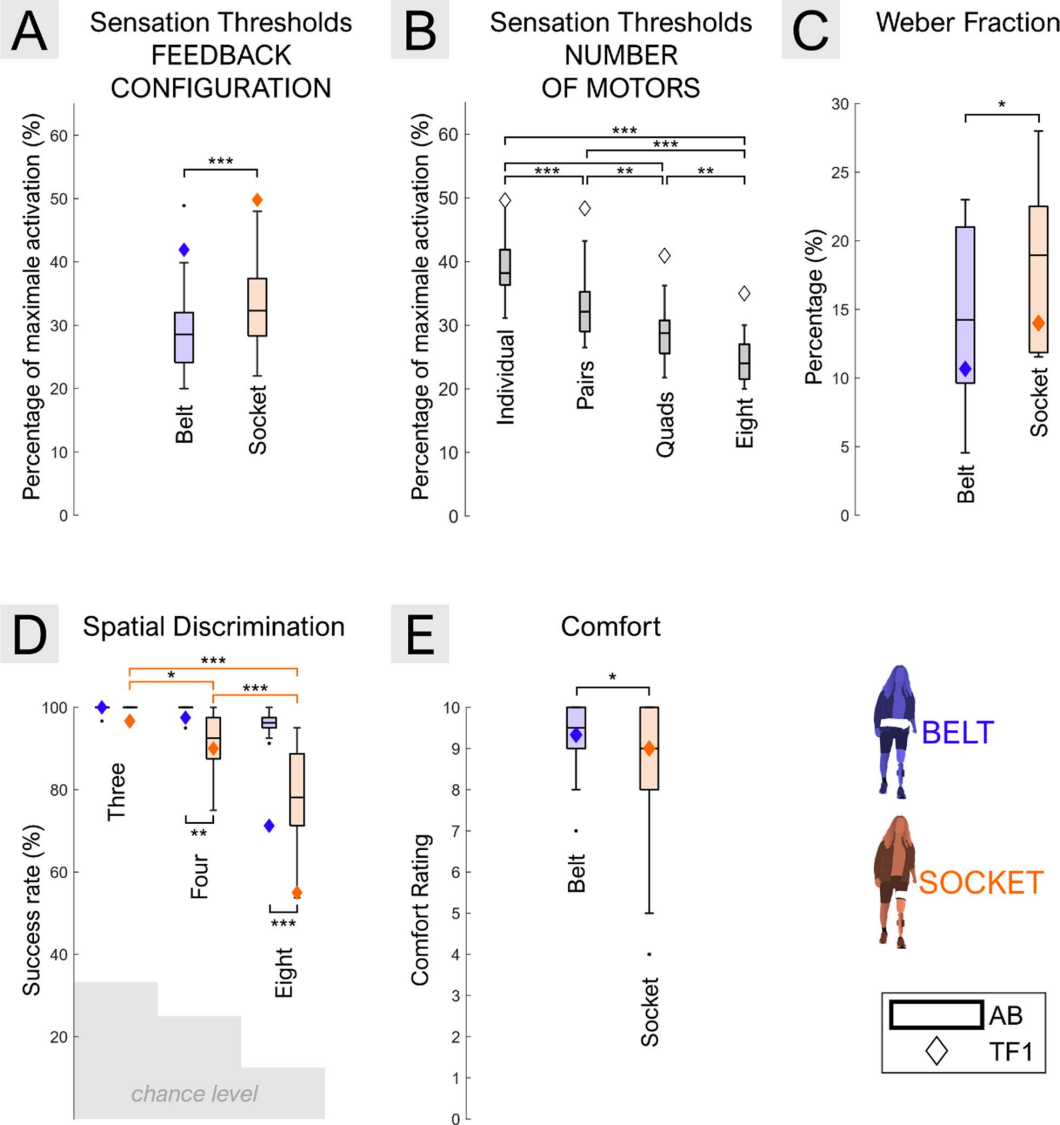
Results of the (**A**, **B**) Sensation Thresholds, (**C**) Weber fraction, (**D**) Spatial Discrimination tests, and (**E**) Comfort evaluation–Sensation thresholds are presented according to (**A**) feedback configuration (i.e., Belt vs. Socket) and (**B**) the number of motors (i.e., Individual, Pairs, Quads and Eight motors). The Weber Fraction (**C**) is shown by feedback configuration, while the spatial discrimination plot (**D**) illustrates the significant interaction between feedback configuration (i.e., Belt vs. Socket) and the number of patterns (i.e., Three, Four, and Eight choices). The comfort plot (**E**) represents subjective ratings of comfort (0 = not comfortable, 10 = very comfortable) for the Belt vs. Socket placement (main effect of feedback configuration). In all plots, blue and orange colors represent the Belt and Socket configurations, respectively. Boxplots summarize data from the 10 able-bodied participants, while the diamond indicates the participant with TFA. Significance levels are indicated as follows: *: p < 0.05; **: p < 0.01; ***: p < 0.001

The results for the Sensation Thresholds (ST) are displayed in Fig. 10 (A, B). In able-bodied participants, there was no significant interaction between the number of active motors and feedback configuration, while the main effects of both factors were significant. Specifically, the ST was significantly higher for the Socket vs. Belt configuration (33.19 ± 6.58% vs. 29.09 ± 6.47%, p < 0.001, Fig. 10–A), suggesting a somewhat reduced sensitivity when the motors were embedded in the socket. Additionally, the ST decreased as the number of active motors increased (p < 0.001, Fig. 10–B). These results suggest that vibrotactile perception inside the socket is generally lower relative to the Belt and that increasing the number of active motors may help improve detection. A similar pattern was observed in the participant with TFA, although with a slight overall shift toward higher thresholds, indicating somewhat reduced sensitivity compared to the able-bodied group.

The results of the Weber Fraction (WF) are shown in Fig. 10–C. In able-bodied participants, the WF was significantly higher for the Socket vs. Belt configuration (17.98 ± 5.72% vs. 14.49 ± 7.02%, p < 0.01), suggesting that the ability to distinguish between different vibration intensities was reduced when the motors were placed inside the socket. Similar trends are seen in the participant with TFA, and the estimated WF is within the range obtained in the able-bodied participants.

The success rates achieved in the Spatial Discrimination (SD) task are displayed in Fig. 10–D. In able-bodied participants, a significant interaction between the number of spatial patterns and feedback configuration was found (p < 0.001). The success rates in the SD task were significantly lower in the Socket vs. Belt configuration with four patterns (99.25 ± 1.69% vs. 91.50 ± 7.66%; p < 0.01) and eight patterns (96.0 ± 2.81% vs. 78.13 ± 12.60%; p < 0.001). However, the performance was similar and nearly perfect (i.e., close to 100%) in both configurations when only three patterns were presented. Notably, the success rate in able-bodied participants remained high across all conditions with the Belt, regardless of the number of patterns presented. In contrast, performance in the Socket configuration declined as the number of options increased, with significant drops between the three vs. four patterns (99.67 ± 1.05% vs. 91.5 ± 7.66%; p < 0.05), three vs. eight (99.67 ± 1.05% vs. 78.13 ± 12.60%; p < 0.001) and four vs. eight (91.50 ± 7.66% vs. 78.13 ± 12.60%; p < 0.001). In the participant with TFA, the results followed the same trends overall, but she showed substantially lower performance compared to the able-bodied participants in the Belt scenario in the task with eight patterns. Yet, her performance in the corresponding Socket configuration was even poorer. Therefore, as the spatial discrimination task becomes more challenging (i.e., more options), the Belt configuration offers a clear advantage over the Socket placement. Even in the most demanding conditions (i.e., identifying individual motors), the Belt configuration enabled able-bodied participants to achieve a median success rate above 80%.

**Fig. 11:**
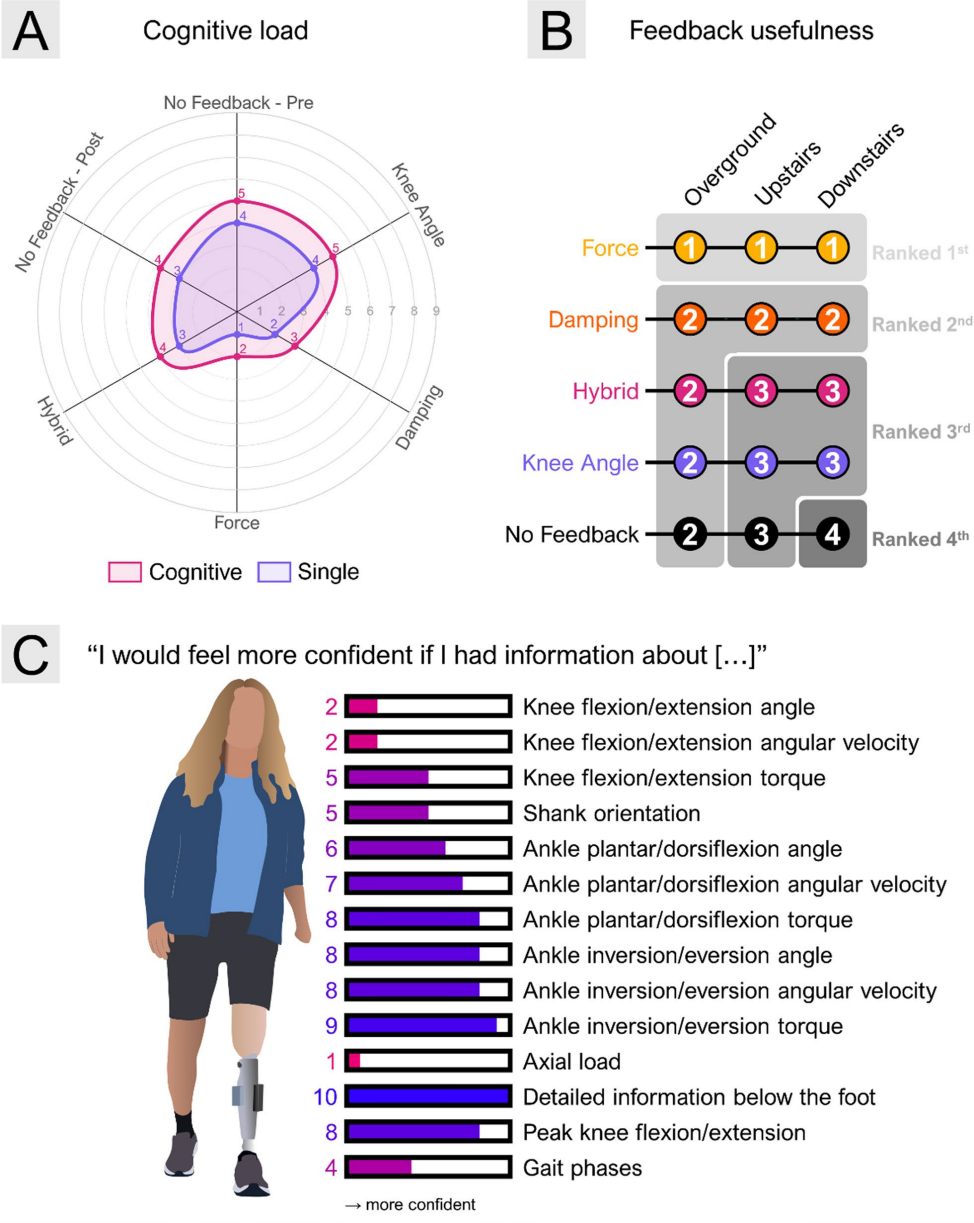
Results of the (**A**) cognitive load evaluation, (**B**) the feedback usefulness, and (**C**) feedback needs questionnaires–The results of the cognitive load evaluation (**A**) are displayed using a radar plot, during walking without (purple) and with a parallel cognitive task (pink) for each feedback condition (no feedback, knee angle, damping, force, and hybrid feedback). The ranking of the usefulness perceived of the different schemes is displayed in (**B**) and is distinguished for overground, upstairs, and downstairs walking. The participant could rank one or multiple feedback schemes at the same level of usefulness. The results of the questionnaire displayed in (**C**) show a rating from 0 to 10 for each feedback scheme that the TFA participant would feel more confident with

The results in the subjective experience of comfort when using the Belt and Socket configurations are shown in Fig. 11. While most participants rated both configurations as generally comfortable, statistical analysis revealed significantly higher comfort scores for the Belt compared to the Socket (9.17 ± 1.02 vs. 8.15 ± 1.96; p < 0.05). Importantly, there was no significant change in comfort over time, suggesting that perceived comfort did not deteriorate with prolonged use. Additionally, neither the interaction between the time of assessment (T1, T2, and T3) and feedback configuration nor the main effect of time reached statistical significance, implying that initial comfort perceptions are stable and that the Belt configuration may offer a more consistently comfortable solution for long-term wear.

### Preliminary out-of-the-lab assessment of feedback schemes

When provided any type of feedback, the participant walked slightly faster during the cognitive task (mean ± SD over all feedback conditions: 1.21 ± 0.03 m/s vs. 1.17 ± 0.03 m/s). In contrast, when no feedback was provided, walking speed remained largely unchanged regardless of the presence of a cognitive task (1.18 ± 0.05 m/s with cognitive task vs. 1.19 ± 0.04 m/s without). The detailed results are provided in Figure S1 in Supplementary Materials.

The symmetry indices of the spatiotemporal parameters (stance time, stance percentage, gait cycle time) and angle kinematics during overground, upstairs, and downstairs walking are reported in Figures S2 and S3 in Supplementary Materials). All measures were consistently high for this participant during overground walking and appeared unaffected by the feedback or dual tasking, indicating the level of symmetry close to that characterizing gait of able-bodied participants [71]. Gait cycle time symmetry was also high during stair climbing (similar to that reported for able-bodied participants [71]) and rather stable across conditions, except for the knee angle feedback during downstairs walking without cognitive task (symmetry decreased with feedback).

The symmetry of stance time and stance percentage were affected by the conditions when walking downstairs and upstairs. The addition of the cognitive task generally decreased these parameters, and the impact of feedback varied depending on the approach and the walking task but remained overall modest (< 10%). During upstairs walking, the feedback did not have a positive effect on the two outcome measures, and in some cases, they even decreased (see Figure S2 in the Supplementary materials). For instance, with knee angle feedback, the stance time symmetry dropped (single task: 72.35 ± 10.70%; cognitive task: 72.86 ± 9.79%) compared to the no feedback condition (single task: 78.87 ± 9.46%; cognitive task: 76.62 ± 9.17%). The same was observed for the stance percentage symmetry (single task: 73.60 ± 9.43%; cognitive task: 73.43 ± 8.54%) compared to no feedback condition (single task: 79.79 ± 8.20%, cognitive task: 76.70 ± 9.02%). Overall, the force feedback maintained the highest symmetry compared to other feedback approaches for both stance time (single task: 78.39 ± 10.67%; cognitive task: 76.50 ± 9.76%) and stance percentage symmetries (single task: 79.14 ± 10.03%; cognitive task: 76.58 ± 8.96%). During downstairs walking, on the contrary, the feedback mostly increased the symmetry. For stance time symmetry, the most consistent improvement was achieved with force feedback (single task: 81.15 ± 7.74%; cognitive task: 76.52 ± 8.92%) and knee angle feedback (single task: 78.72 ± 14.33%; cognitive task: 80.15 ± 7.38%) compared to no feedback (single task: 75.93 ± 9.50%; cognitive task: 72.98 ± 9.09%). Similarly, the stance percentage symmetry increased with force feedback (single task: 82.98 ± 2.97%; cognitive task: 78.53 ± 6.34%) and knee angle feedback (single task: 89.34 ± 8.11%; cognitive task: 80.21 ± 5.95%) compared to no feedback (single task: 78.12 ± 5.37%; cognitive task: 76.16 ± 6.77%). However, knee angle feedback also negatively impacted the gait cycle time symmetry (single task: 85.24 ± 9.43%; cognitive task: 96.14 ± 2.77%) during downstairs walking compared to no feedback during the single task (single task: 95.28 ± 3.58%; cognitive task: 95.05 ± 3.69%).

These findings suggest that force feedback may best support the user’s ability to maintain symmetrical loading and timing during more demanding locomotor tasks such as stair descent, as seen by the most consistent positive impact overall across walking tasks (either no change or improved symmetry). Nevertheless, these changes remain modest relative to the within-condition variability, indicating that the observed differences should be interpreted with caution.

Regarding overall gait kinematics, it did not appear to show any clear trend except for the pelvis tilt symmetry, which seemed reduced during dual-task overground walking regardless of feedback presence (58.48 ± 3.21% across all conditions with cognitive task vs. 68.24 ± 3.09% without), suggesting that the added cognitive task may have had a subtle effect on gait coordination.

Overall, the participant with TFA rated the conditions with the parallel cognitive task as more cognitively demanding (Fig. 11–A). Importantly, when walking with force and damping feedback, the reported cognitive load was reduced (ratings of 2 and 3, respectively), whereas walking with knee angle or hybrid feedback resulted in higher cognitive load ratings (4 and 5), similar to walking without feedback. These findings suggest a potential effect of specific types of feedback, particularly those related to force or damping, in contributing to offload cognitive demands during dual-task walking.

As shown in Fig. 11 (B), the participant rated the force feedback as being the most useful across all walking tasks, followed by damping feedback. This aligns with the observed reduction in cognitive load during those conditions. The knee angle and hybrid feedback were rated the same as the damping in overground walking, but their perceived usefulness decreased in the upstairs and downstairs scenarios. Importantly, in stair descent, any form of feedback was perceived as more useful than none, while in overground walking, only force feedback was perceived as more useful than no feedback. These patterns may indicate that certain feedback modalities may be more beneficial in scenarios with higher physical and cognitive demands.

When asked to evaluate the importance of various feedback variables (Fig. 11–C), the participant rated the force profile beneath the foot, along with ankle plantarflexion/dorsiflexion and inversion/eversion angle, as the most valuable. In contrast, feedback about the knee angle, gait phases, and axial load was considered less critical. This preference closely mirrors the usefulness rankings of the tested feedback schemes, where the participant ranked knee angle and hybrid feedback lower than damping and force feedback. The participant explained that she could already perceive the axial load through the socket, but lacked information about the distribution of the force under the insole. This perspective was reflected in her ratings, with axial load receiving a 1/10 and insole force distribution a 10/10. These insights suggest that the perceived relevance of feedback might be influenced by the participant’s residual sensory capabilities and specific informational needs, which are likely to vary across users and should be carefully considered when designing feedback systems.

## Discussion

Non-invasive artificial sensory feedback techniques allow for providing information from the prosthesis, using low-cost and low-energy solutions that can be embedded inside the socket. Focusing on such technology might have a great impact on the lives of people with lower-limb amputation, as the approach is straightforward to implement, and there is no need for additional surgeries, which are required when using invasive solutions. However, interfacing feedback systems directly with MP prostheses (knee and ankle) is rarely presented, and the comparison between multiple configurations and schemes in terms of placement and feedback variables is seldom conducted.

In the current study, we developed a self-contained framework comprising the main controller communicating with the prosthesis and feedback system to implement online mapping of the information from the embedded sensors into stimulation profiles delivered to the user. Importantly, the mapping can be configured, selected, and downloaded from the laptop to the controller, enabling fast prototyping and flexible testing of different encoding schemes. We used the system to compare the two feedback configurations (Belt and Socket) during treadmill walking. We then leveraged the fact that the system is self-contained and easily configurable to test four feedback methods in an ecological scenario, outside of the lab.

In the first part of the study, comparing the spatial and amplitude perception as well as overall comfort between the Belt and Socket configurations in able-bodied participants revealed a clear trade-off between the portability and the perceptual clarity. While the Socket configuration represents a more compact and self-contained solution, the Belt setup, through the addition of an additional wearable, results in improved perception performance.

The consistently smaller ST and WF in able-bodied participants when using the Belt configuration indicates a more sensitive and higher-fidelity perception compared to the Socket. This suggests that the Belt configuration could provide higher-resolution feedback, particularly when amplitude modulation is used to convey the information. While prior studies suggest that both the waist and thigh have relatively low amplitude perception capacities [74–76], several factors could explain the improved performance of the Belt over the Socket. These include sensory gating effects [64, 77] but also influences such as socket pressure, skin stretches, and muscle movements inside the socket during walking. Importantly, our findings align with previous work [78], reporting slower reaction times to vibrotactile stimuli at the thigh compared to the waist during walking. This suggests that anatomical site-specific differences in sensory information processing may further contribute to the observed effects. Interestingly, the results for the participant with TFA appear to follow the same trends as those of the able-bodied group, but her perception was also consistently worse in both Socket and Belt configurations. While the difference in the material (silicon liner for the socket and foam for the bypass system) and the setup/attachment to the leg/residual limb could explain lower performance with the Socket placement, the worse perception in the Belt configuration implies that the results also reflect the general intersubject differences, which are particularly expressed in people with lower-limb amputation [33]. Another important aspect to underline is that the participant with lower-limb amputation walked faster than the able-bodied participants, which may have further affected sensory perception and contributed to the observed shift in subjective experience.

The conclusions are similar regarding the spatial perception capacities in able-bodied participants and the participants with lower-limb amputation, but the difference between the Belt and Socket becomes pronounced when the task difficulty increases. When the feedback encoding is simple, as when the task was to discriminate three patterns, both configurations led to a high performance. However, better performance of the Belt placement appears for higher number of patterns (e.g., identification of individual motors), which can be explained by the larger distance between the motors, and the presence of anatomical landmarks (navel, spine), which could altogether help in identifying the location of the stimulus [76].

The overall conclusion, based on amplitude and spatial perception results, is that the Belt configuration can be considered when developing, testing, and using feedback with complex encoding. For instance, this could be feedback based on amplitude modulation, where the high resolution (many discriminable levels) is important, or when encoding many events/states using distinct spatial patterns. Other studies used Belt placement to provide feedback with promising results, but they did not compare the Belt to the Socket configurations [41, 65, 79, 80]. Overall, this shows that the Belt is a suitable approach to providing feedback, but the present study also demonstrates that when carefully designed (e.g., by reducing the number of options), this feedback can also be embedded directly inside the socket. Although the Belt showed promising results and was reported as comfortable, further studies are needed to determine whether its potential benefits outweigh the inconvenience of adding a component external to the prosthesis. It is especially relevant to extend this analysis to more participants with lower-limb amputation, as the slightly worse results obtained in this participant might suggest worse perception capacities and interindividual differences in these participants, potentially indicating the necessity to tailor the feedback to the needs and capacities of a specific user.

In the second part of the study, we used the Socket configuration of the flexible feedback-prototyping system to assess the impact of four feedback schemes on the user's experience, cognitive load, and biomechanics. Overall, a slight increase in overground walking speed was observed during dual-tasking conditions when feedback was provided (1.21 ± 0.03 m/s vs. 1.17 ± 0.03 m/s). More importantly, force feedback appeared to be a potentially more promising strategy, as it led to greater improvements or preservation of spatiotemporal symmetry, compared to other feedback methods especially during stair ascent and descent (see Results section). Additionally, the participant found this feedback more useful compared to the three other approaches, indicating lower cognitive effort and increased confidence when using it.

Knee angle and gait phases are often considered as the feedback variables in the literature, and the studies report improvements in gait kinetics and kinematics when such feedback is provided non-invasively [18–20, 43, 50, 52]. However, these results are often obtained during treadmill walking [18, 20, 50, 52], walking overground but over short distances [19, 43], and/or by focusing on specialized tasks where the participant is additionally challenged to promote the reliance on feedback (e.g., walking over an uneven terrain [20], blocking vision [81]). Even in such cases, the benefits of feedback are often small, and it is unclear if the impact is also clinically significant [20]. In this study, the subjective ratings by the user indicated that knee angle and hybrid feedback were not experienced as relevant regarding the decrease in cognitive load as well as more generally (Fig. 11). In our experiment, the participant performed the typical daily life ambulatory tasks while receiving full incidental feedback as usual during prosthesis use, and she specifically indicated that she already receives this information from incidental sources. Instead, she mentioned that she would prefer the feedback informing her about the state of the more distal segments of the prosthesis. The most useful feedback would help her understand what is happening below the foot, for instance, if the foot is slipping or if the ankle is not positioned properly. Interestingly, she highly rated the inversion/eversion angle around the ankle, as she mentioned that this is critical for the stability of the current step as well as subsequent adaptation during the gait cycle. This feedback variable however has not been considered yet in the literature. Importantly, this participant appeared to benefit from the force feedback in our first experiment [59], which aligns with the benefits expressed in this experiment. These findings therefore suggest that higher task complexity may heighten the need for external support beyond incidental feedback, as seen for benefits observed during stair ambulation compared to overground walking. The damping feedback was the second most highly rated approach, and was perceived as particularly useful in the upstairs/downstairs scenario compared to more commonly used feedback approaches (knee angle). The likely reason is that this approach conveys information relevant to knee stability, which is difficult to estimate from the incidental sources because the damping is adjusted internally by the prosthesis through mechanisms that are not fully transparent to the user. In that sense, this approach has a similar idea to the concept of EMG biofeedback that was successfully demonstrated in upper limb prosthetics, but without the active control of the prosthesis angular motion [60, 62]. The damping feedback allows the participant with amputation to anticipate when the leg is ready for weight acceptance, which is especially important when performing stair ascent and descent. The results in the present study suggest that the combination of embedded (damping feedback) and external sensors (insole feedback) could offer complementary benefits, but this needs to be investigated explicitly.

Nevertheless, the improvements observed in spatiotemporal parameters in some conditions with feedback remain modest, and the impact of any feedback type on kinematics symmetry was minimal. First, although the aim was to demonstrate the usability of the developed framework, a clear limitation of the present study is that the out-of-the-lab assessment of the feedback system was conducted with only a single participant with TFA. As this individual demonstrated a high level of functional ability and may not be representative of the wider population with amputation, the findings should be interpreted with caution. Second, this might be explained by the fact that this participant showed high levels of symmetry (especially during overground walking), which may have limited the observable benefits of the feedback. It is possible that the feedback might express more benefits in novice participants rather than experienced users, as provided sensory information may play a more prominent role in facilitating the learning to walk with a prosthesis. Third, the variability of the spatiotemporal parameters was high within a trial, particularly during stair ascent and descent. This variability likely stemmed from differences in overall cycle time and the participant’s inability to maintain a stable rhythmic pattern while negotiating stairs. The variability within trials often exceeded or was comparable to the changes observed between the feedback conditions, suggesting that the observed differences in symmetry indices may reflect both condition-dependent effects and trial-dependent fluctuations. Fourth, a potential factor would be the limited training time and exposure to each feedback modality, which may not have allowed sufficient adaptation or learning. This study contrasts with other studies that have demonstrated benefits after prolonged or repeated exposure to feedback [43, 50]. This is the consequence of the tradeoff between testing a single feedback modality (over prolonged time) or multiple modalities, which is an interesting point to further explore in future work. Fifth, we selected the Socket configuration because this is a self-contained solution, as both Belt and Socket placement were rated as comfortable by the participants, and the tested feedback schemes used simple spatial encoding with 3 and 4 patterns (where both configurations performed well). Therefore, it is unlikely that the performance would be substantially different with the Belt placement for the damping and gait phase feedback since the encoding was simple and easily discriminable (as shown by the results of the psychometric test). However, for the knee angle and especially force feedback, it is conceivable that functional performance and cognitive load might improve further when using the Belt configuration, which remains to be tested in future work.

## Conclusion

This initial investigation offers important insights into feedback design and evaluation in ecological scenarios. First, the results show that the decision about where to place the motors, in the socket versus externally (e.g., belt), should consider the complexity of the feedback encoding. Second, the benefits and perceived utility of different feedback variables may depend on user preferences and the extent to which similar information is already available to the user through incidental cues (e.g., socket pressure). Third, there is value in complementing controlled laboratory assessments with testing in ecologically valid settings, where users interact with real-world environments and naturally engage their full range of sensory inputs, as this may better reveal the practical relevance and limitations of feedback strategies. Finally, the participant’s request for ankle inversion/eversion feedback (a variable rarely addressed in current literature), underscores the importance of user-oriented design. Developing novel feedback modalities (such as those based on internal damping or omnidirectional force) beyond commonly explored variables like knee angle or gait phase feedback should receive greater attention, particularly when grounded in explicit user preferences and task-specific demands, as early indications suggest they may be more effective in reducing cognitive effort and increasing confidence. Overall, the preliminary results demonstrate that MP prosthesis-integrated sensors can be utilized not only for gait optimization but also to create compact, meaningful, and personalized feedback solutions. These solutions, when designed in collaboration with the users and validated in real-world conditions, hold strong promise for enhancing prosthesis usability, safety, and user satisfaction. Future work should expand on this framework to assess the applicability across diverse populations of people with amputation, multiple training sessions, and a broader range of daily activities.

## Supplementary Information

Supplementary Material 1.

## Data Availability

The data supporting the findings of this study are readily available within the article and its supplementary materials. Further data are available from the corresponding author, SD, upon reasonable request.
