# Supplementary material for: Development and assessment of vibrotactile feedback from the embedded sensors of a microprocessor-controlled knee prosthesis
Source: J Neuroeng Rehabil. 2025 Dec 19;23:33. doi: 10.1186/s12984-025-01793-8 (PMC12831381; doi:10.1186/s12984-025-01793-8)
Supplement: Supplementary file 1 — Supplementary Material 1. [file 12984_2025_1793_MOESM1_ESM.docx]

**Supplementary Materials**

Development and assessment of vibrotactile feedback from the embedded sensors of a microprocessor-controlled knee prosthesis

Valette Romain ^1^, Gonzalez-Vargas Jose ^2^, Dosen, Strahinja ^1^

^1^ Department of Health Science and Technology, Aalborg University, Aalborg, Denmark.

^2^ Ottobock SE & Co. KGaA, Duderstadt, Germany

Corresponding author: Strahinja Dosen ([sdosen@hst.aau.dk](mailto:sdosen@hst.aau.dk)), Department of Health Science and Technlogy, Aalborg University, Aalborg, Denmark.

| Table S1 – General characteristics of the able-bodied participants | Page 2 |
| --- | --- |
| Table S2 – Characteristics of the participant with transfemoral amputation | Page 2 |
| Figure S1 – Walking speed on overground section | Page 3 |
| Figure S2 – Spatiotemporal parameters symmetry | Page 3 |
| Figure S3 – Kinematics symmetry | Page 4 |

**Table S1 – General characteristics of the able-bodied participants**

| ID | AB1 | AB2 | AB3 | AB4 | AB5 | AB6 | AB7 | AB8 | AB9 | AB10 |
| --- | --- | --- | --- | --- | --- | --- | --- | --- | --- | --- |
| Age (years) | 23 | 35 | 24 | 26 | 24 | 28 | 24 | 23 | 30 | 31 |
| Sex | ♂ | ♂ | ♀ | ♂ | ♂ | ♂ | ♂ | ♀ | ♂ | ♂ |
| Weight (kg) | 81 | 77.1 | 75 | 74 | 73 | 70 | 81 | 62 | 81 | 70 |
| Height (cm) | 178 | 183 | 185 | 178 | 174 | 181 | 190 | 176 | 181 | 185 |
| Foot size (EU) | 43 | 43 | 41 | 42 | 42 | 41 | 46 | 41 | 43 | 45 |
| Leg circumference (cm) | 56 | 53 | 56 | 51 | 55 | 50 | 53 | 52 | 53 | 50 |
| Waist circumference (cm) | 91 | 84 | 86 | 80 | 87 | 79 | 80 | 73 | 88 | 80 |
| Self-selected walking speed (m/s) | 0.58 | 0.53 | 0.50 | 0.56 | 0.47 | 0.47 | 0.64 | 0.44 | 0.56 | 0.50 |

**Table S2 – Characteristics of the participant with transfemoral amputation**

| ID | TFA |
| --- | --- |
| Age (years) | 58 |
| Sex | ♀ |
| Weight (kg) | 65 |
| Height (cm) | 170 |
| Level of autonomy | Independent walker |
| Prosthesis use | Daily |
| Amputation type | Transfemoral |
| Prosthesis side | Left |
| Foot size (EU) | 40 |
| Etiology | Congenital |
| Phantom pain | None |
| Phantom sensation | None |
| Residual limb length (cm) | 19 |
| Personal knee prosthesis | Rheo Knee XC |
| Personal ankle-foot module | Proprio Foot |
| Socket | Northwestern |
| Suspension | Vacuum-assisted socket |
| Tested knee prosthesis | C-Leg 4 |
| Tested ankle-foot module | Pro-Flex XC |
| Residual limb circumference (cm) | 39 |
| Waist circumference (cm) | 74 |
| Self-selected walking speed (m/s) | 0.75 |


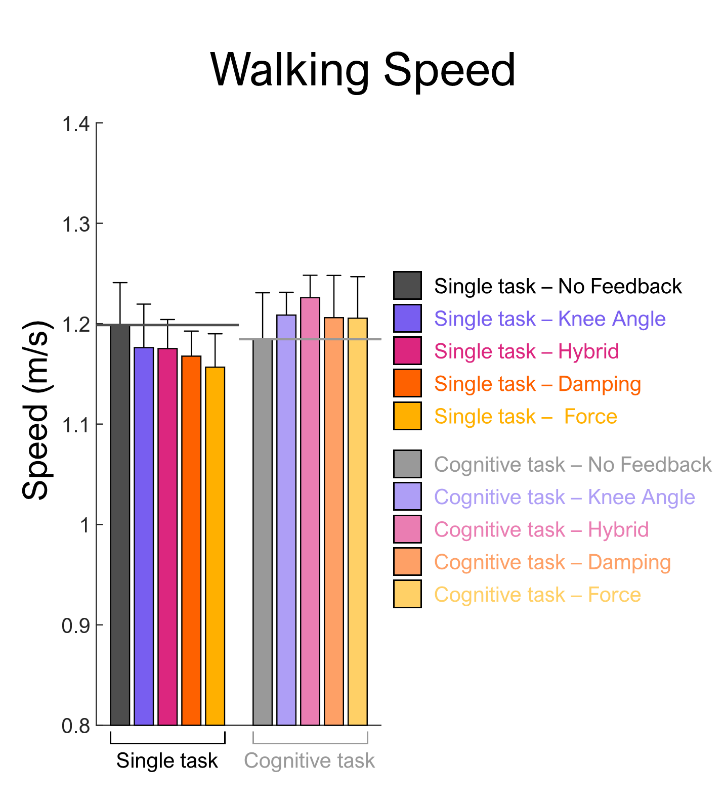


**Figure S1 – Walking speed –** Summary results of the walking speed of the participant with transfemoral amputation in overground walking – while receiving feedback or not, while performing a cognitive task or not. In this plot, the grey bars represent the no feedback conditions, and the colored bars represent the feedback conditions. The darker shades indicate the conditions in single task, and the lighter shades indicate the conditions with cognitive task.


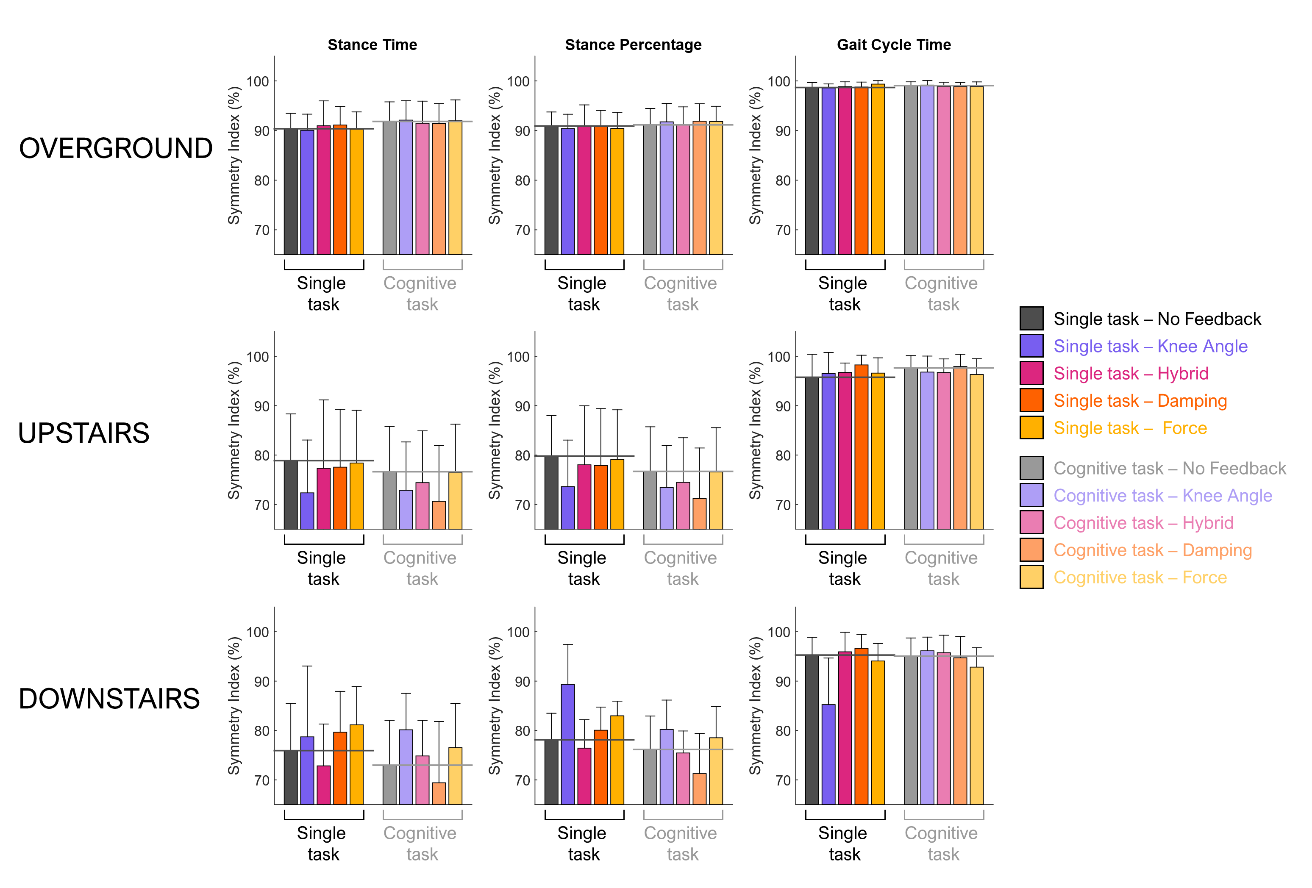


**Figure S2 – Spatiotemporal parameters symmetry –** Summary results of the Symmetry Index of spatiotemporal parameters of the participant with transfemoral amputation in overground, upstairs and downstairs walking – while receiving feedback or not, while performing a cognitive task or not. In all the plots, a Symmetry Index of 100% indicates a perfect symmetry of the gait temporal features, while 0% represents complete asymmetry. In these plots, the grey bars represent the no feedback conditions, and the colored bars represent the feedback conditions. The darker shades indicate the conditions in single task, and the lighter shades indicate the conditions with cognitive task.


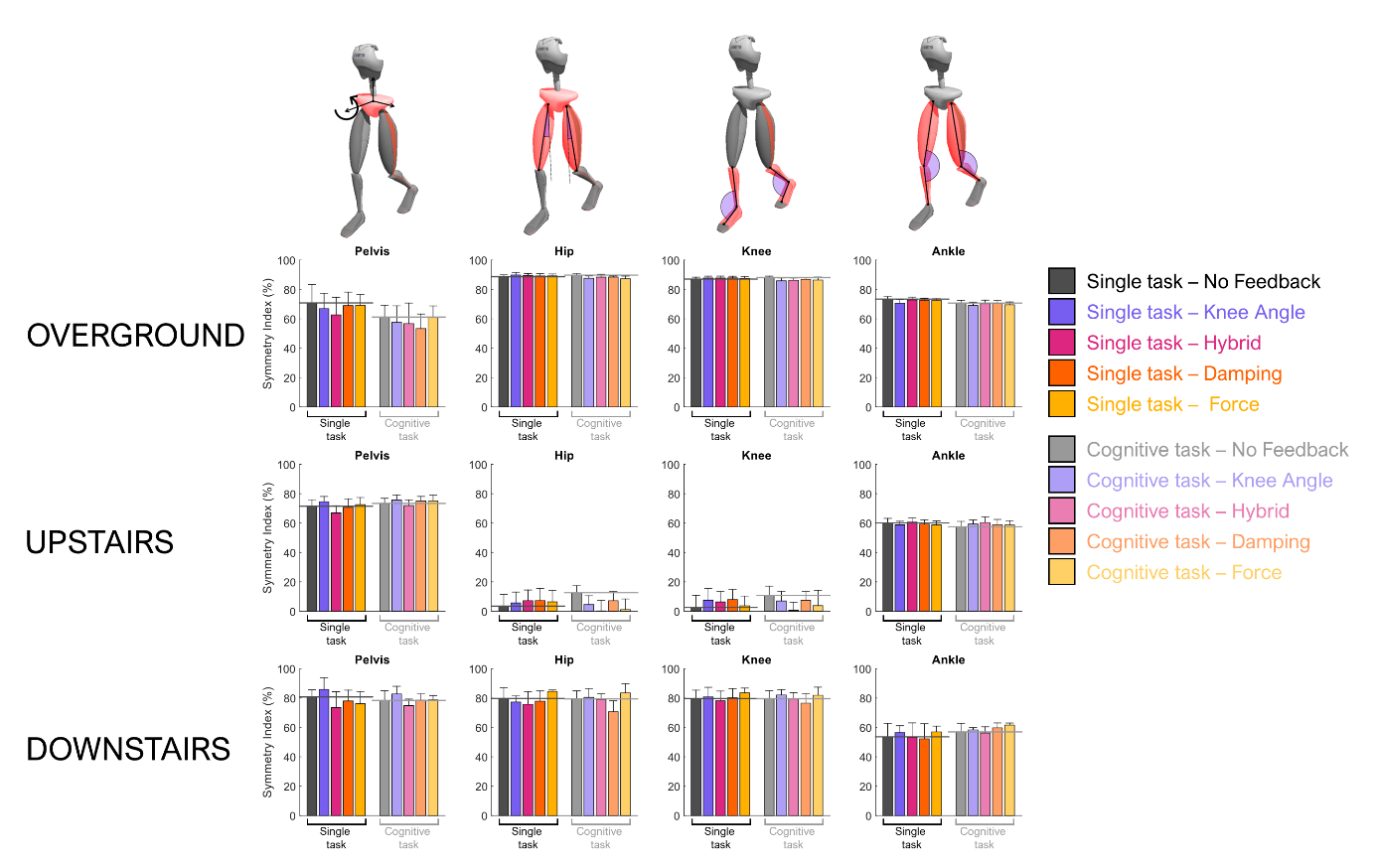


**Figure S3 – Kinematics symmetry –** Summary results of the Symmetry Index on the kinematics of the pelvis tilt, hip, knee and ankle angles of the participant with transfemoral amputation in overground, upstairs and downstairs sections – while receiving feedback or not, while performing a cognitive task or not. In all the plots, a Symmetry Index of 100% indicates a perfect symmetry of the entire gait cycle kinematics, while 0% represents complete asymmetry. In these plots, the grey bars represent the no feedback conditions, and the colored bars represent the feedback conditions. The darker shades indicate the conditions in single task, and the lighter shades indicate the conditions with cognitive task.
